# Molecular Interlayer for High‐Performance and Stable 2D Tin Halide Perovskite Transistor

**DOI:** 10.1002/advs.202409088

**Published:** 2025-04-09

**Authors:** Bum Ho Jeong, Juan Anthony Prayogo, Jongmin Lee, Seok Woo Lee, Dong Ryeol Whang, Dong Wook Chang, Hui Joon Park

**Affiliations:** ^1^ Department of Organic and Nano Engineering & Human‐Tech Convergence Program Hanyang University Seoul 04763 Republic of Korea; ^2^ Department of Industrial Chemistry and CECS Research Institute Pukyong University Busan 48513 Republic of Korea; ^3^ Department of Advanced Materials Hannam University Daejeon 34054 Republic of Korea; ^4^ Department of Semiconductor Engineering Hanyang University Seoul 04763 Republic of Korea

**Keywords:** 2D tin halide perovskite, dipole moment, energy barrier reduction, field‐effect transistor, stability, surface passivation

## Abstract

Tin (Sn) halide perovskites present considerable potential for the advancement of high‐performance p‐channel field‐effect transistors (FETs), attributable to their low hole effective mass and reduced carrier scattering. However, their intrinsic instability has impeded their ability to achieve the anticipated performance benchmarks. In this study, molecular interlayers are designed that not only passivate surface defects in Sn perovskites through their functional groups, leading to improved film formation and consequently enhanced performance and stability but also reduce the energy barrier at the source and drain interfaces through their strong dipole moments, thereby enhancing carrier transport. These synergistic effects result in FET devices exhibiting remarkable performance metrics, including effective mobility exceeding 11 cm^2^ V^−1^ s^−1^ and an on/off ratio greater than 1.3 × 10^7^ while securing exceptional durability and reproducibility. Furthermore, the hydrophobic characteristics of the surface interlayer confer superior storage stability.

## Graphical abstract

Molecular interlayers are designed for p‐channel 2D Sn halide thin‐film transistors, yielding exceptional performance metrics, a significant reduction in hysteresis, and enhanced ambient stability. The effectiveness of new molecular interlayers (TPOF1, TPOF2, and 2F‐TOPF2) is confirmed by surface treatment, demonstrating remarkable carrier mobility (µ_claimed_ > 14 cm^2^ V^−1^ s^−1^) and on/off ratio (*I*_on_/*I*_off_ > 10^7^), while securing noteworthy durability and reproducibility.

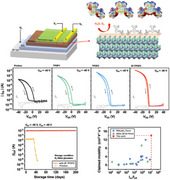

## 1 Introduction

Metal halide perovskites (MHPs) have rapidly emerged as a leading class of semiconductor materials due to their exceptional optoelectronic properties, including long carrier diffusion length, tunable bandgap, and high absorption coefficient, as well as their facile solution‐processed fabrication.^[^ ^1^, ^2^, ^3^ ^]^ These attributes have driven unprecedented performance and cost‐effectiveness in various devices such as solar cells, light‐emitting diodes (LEDs), detectors, and lasers.^[^ ^4^, ^5^, ^6^, ^7^, ^8^, ^9^, ^10^ ^]^ However, despite these advancements, the utilization of MHPs in field‐effect transistors (FETs) remains relatively underexplored and merits further attention.^[^ ^11^, ^12^, ^13^, ^14^ ^]^ This research endeavor is crucial as it offers a platform for gaining deeper insights into the carrier transport dynamics and the physical properties of the perovskites under diverse operational conditions, which is essential for advancing optoelectronic devices. Furthermore, this exploration can address the ongoing challenge of identifying high‐performance channel materials for FET devices that are also amenable to facile processing.

In the initial development of perovskite FETs, research primarily focused on lead (Pb)‐based 3D perovskite systems, such as methylammonium lead iodide (MAPbI_3_).^[^ ^15^, ^16^, ^17^ ^]^ However, the substitution of Pb with tin (Sn), which has a similar ionic radius (1.18 Å for Sn, 1.19 Å for Pb), has shown promise in enhancing charge carrier mobility at room temperature, because Sn‐based perovskite systems exhibit a smaller hole effective mass and reduced carrier scattering due to weaker Fröhlich interactions.^[^ ^18^, ^19^, ^20^ ^]^ This approach holds significant potential for implementing high‐performance p‐channel FETs, which currently lag behind n‐channel devices in complementary circuit development.^[^ ^21^ ^]^ Moreover, the Sn‐based systems can be environmentally friendly alternatives to the Pb‐based systems.^[^ ^22^, ^23^ ^]^

However, 3D perovskite systems are sensitive to ionic defects, which often impede charge transport behavior due to extensive ion migration at room temperature.^[^ ^24^, ^25^ ^]^ Those mobile ions can also screen the applied gate electric field, reducing the gate modulation of the current and yielding low field‐effect mobility and large hysteresis during device operation. Therefore, 3D perovskite‐based transistors are often operated in ion suppression environments, such as low temperatures.^[^ ^26^ ^]^ To address these issues, small organic cations (e.g., methylammonium (MA^+^) and formamidinium (FA^+^)) in 3D perovskite structures can be replaced with large organic cations such as phenethylammonium (PEA^+^) and butylammonium (BA^+^) to form 2D perovskite structures, which can mitigate the deleterious effects of ion migration due to their layered structure.^[^ ^27^ ^]^ These 2D perovskite systems feature alternating layers composed of continuous corner‐sharing metal‐halide octahedral inorganic layers and bulky organic cation layers, possessing a highly ordered crystal structure.^[^ ^28^ ^]^ Due to the hydrophobicity nature of the bulk organic cations, their stability in ambient conditions is also enhanced. Consequently, these 2D structures are considered promising candidates for the channel layer of FETs.

Among the 2D Sn halide perovskites, the 2D Ruddlesden‐Popper (RP)‐phase, such as phenethylammonium tin iodide (PEA_2_SnI_4_), has been investigated as one of the most common compositions for FETs.^[^ ^29^ ^]^ However, despite its enhanced stability, these 2D Sn perovskites still suffer from easy oxidation of Sn^2+^ to its tetravalent state Sn^4+^, resulting in ionic defects and unintended p‐type self‐doping.^[^ ^30^, ^31^ ^]^ Furthermore, the relatively rapid crystallization rate of Sn‐based perovskites compared to Pb counterparts leads to low surface coverage and poor film morphology, resulting in a high defect density.^[^ ^32^ ^]^ Additionally, the directly contacted interface between the perovskite channel layer and metal contacts is prone to inducing a Schottky barrier, and this is expected to be more pronounced in the Sn‐perovskite system having heavy p‐doping, resulting in a deeper Fermi energy level (*E*_F_).

In this work, we newly designed molecular interlayers capable of passivating surface defects in 2D RP PEA_2_SnI_4_ perovskite, forming Lewis adducts. Furthermore, these molecules are engineered to exhibit high intrinsic dipole moments, creating a dipole layer at the interface between the perovskite and metal contact. This dipole layer induces ohmic contact by reducing the work function of the perovskite, thereby lowering the contact resistance. Following the surface treatment, the optimized p‐channel PEA_2_SnI_4_‐based FETs exhibit high carrier mobility, achieving outstanding metrics (with µ_claimed_ up to 14.08 cm^2^ V^−1^ s^−1^ and µ_effective_ up to 11.32 cm^2^ V^−1^ s^−1^, as detailed in Note S1, Supporting Information) and a notable suppression of the hysteresis. Additionally, hydrophobic fluorine functional groups, introduced into the molecules to adjust their dipole moment and energy level, significantly enhance the ambient stability of the devices against moisture degradation.

## 2 Results and Discussion

### 2.1 Design of Molecular Interlayers

**Figure 1:**
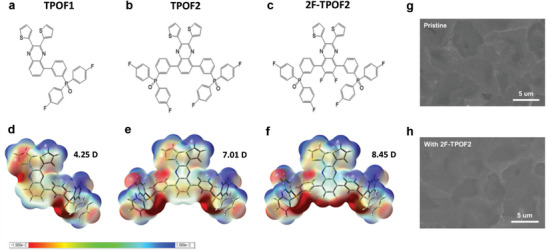
a–c) Chemical structures and d–f) molecular electrostatic potentials of the designed molecules: (a,d) TPOF1, (b,e) TPOF2, and (c,f) 2F‐TPOF2. Theoretically calculated dipole moment values are added to (d–f). g,h) SEM images of top view PEA_2_SnI_4_ films (scale bar: 5 um): (g) pristine and (h) with 2F‐TPOF2.

**Figure** **1**a–c depict the designed molecular structures: (3‐(2,3‐Di(thiophen‐2‐yl)quinoxalin‐5‐yl)phenyl)bis(4‐fluorophenyl)phosphine oxide, (2,3‐Di(thiophen‐2‐yl)quinoxaline‐5,8‐diyl)bis(3,1‐phenylene))bis(bis(4‐fluorophenyl)phosphine oxide), and ((6,7‐Difluoro‐2,3‐di(thiophen‐2‐yl)quinoxaline‐5,8‐diyl)bis(3,1‐phenylene))bis(bis(4‐fluorophenyl)phosphine oxide), denoted as TPOF1, TPOF2, and 2F‐TPOF2, respectively. The experimental synthetic procedures for these molecules are described in the Experimental Section and Figure S1 (Supporting Information). Detailed characterization of the target molecules, including ^1^H, ^13^C, ^19^F, and ^31^P nuclear magnetic resonance (NMR) spectroscopy, mass spectroscopy (MS), and thermogravimetric analysis (TA), is provided in Figures S2–S17 (Supporting Information).

In the synthesis of quinoxaline‐phosphine oxide‐based small molecules, a thienyl‐substituted quinoxaline core was selected over its phenyl counterpart for its ability to enhance intermolecular interactions, particularly through π–π stacking, due to the presence of sulfur atoms in the thienyl rings, which promote stronger molecular packing.^[^ ^33^, ^34^ ^]^ This improved packing facilitates more efficient charge transport and also enhances the effectiveness of the passivation layer in promoting charge transfer across the perovskite and metal contact interface, reducing non‐radiative recombination and contributing to overall device performance.^[^ ^35^, ^36^, ^37^ ^]^ Additionally, the incorporation of a phosphine oxide (P═O) group, decorated with strong electron‐withdrawing fluorine atoms, into the quinoxaline core was aimed at inducing a strong molecular dipole moment and providing passivation effects on perovskite films.^[^ ^38^, ^39^ ^]^ The P═O unit efficiently coordinates with halogen vacancy defects in Sn perovskites through the oxygen (O) atom functioning as a Lewis base^[^ ^40^, ^41^ ^]^ and interacts with Sn^2+^, mitigating their oxidation,^[^ ^40^ ^]^ ultimately enhancing the quality of perovskite films. This enhancement promotes efficient charge transport and improved stability, as discussed in detail later. Moreover, these molecules exhibit good solubility in alcohols, which are recognized as orthogonal solvents suitable for their casting onto perovskite films.^[^ ^42^, ^43^, ^44^, ^45^, ^46^ ^]^

To tune the molecular dipole moment, the *para*‐fluorinated triphenylphosphine oxide unit was introduced either on one side (5‐position) or two sides (5‐ and 8‐positions) of the quinoxaline core, leading to TPOF1 or TPOF2, respectively. Furthermore, two fluorine atoms were additionally attached to the 6‐ and 7‐positions of the quinoxaline core in the TPOF2 to further enhance the molecular dipole moment, resulting in 2F‐TPOF2. The larger number of fluorine substituents not only enhances the molecular dipole moment, but also improves the hydrophobic nature of the molecules, which are favorable for blocking oxygen and moisture.^[^ ^47^, ^48^, ^49^ ^]^ These elevated dipole moments increase the Fermi energy level of the perovskite, inducing ohmic contact with Au and thus reducing the contact resistance, and the enhanced hydrophobic properties maximize the ambient stability. We will also discuss these positive effects in detail later.

The dipole moment values of the molecules (TPOF1, TPOF2, and 2F‐TPOF2), along with their optimized geometries and molecular frontier orbitals, are predicted by density functional theory (DFT) calculations using the Gaussian model at the B3LYP/6‐31G^**^ level (Figure 1d–f; Figure S18, Supporting Information).^[^ ^50^ ^]^ As depicted in Figure S18 (Supporting Information), across all synthesized small molecules, the electron density of the highest occupied molecular orbital (HOMO) and lowest unoccupied molecular orbital (LUMO) predominantly localized over the electron‐donating thiophene rings and the electron‐withdrawing quinoxaline unit. The calculated dipole moments for TPOF1, TPOF2, and 2F‐TPOF2 gradually increased to 4.25 D, 7.01 D, and 8.45 D, respectively, as expected. For reference, the theoretical energy levels of the HOMO/LUMO for TPOF1, TPOF2, and 2F‐TPOF2, estimated by DFT calculations, are 5.87/−2.17 eV, 5.92/−2.29 eV, and 5.99/−2.38 eV, respectively (Figure S18, Supporting Information). This trend is consistent with the experimental values determined by UV–vis spectroscopy and cyclic voltammetry (CV) (Figures S19 and S20, Supporting Information), which are summarized in Table S1 (Supporting Information).

### 2.2 Passivation of PEA_2_SnI_4_ Films with Molecular Interlayer

**Figure 2:**
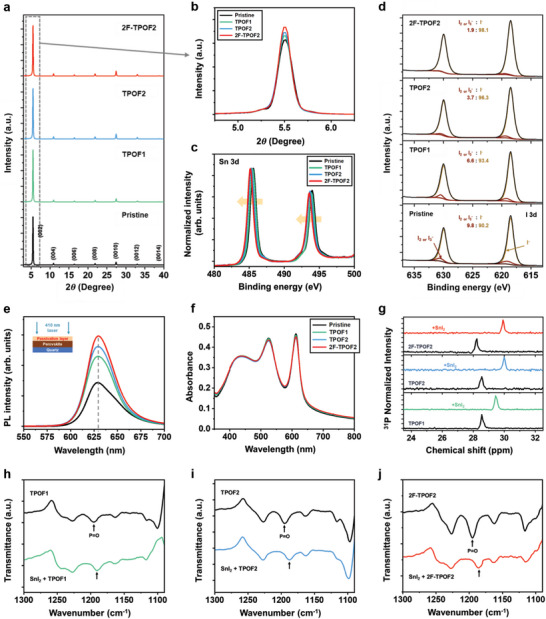
a) XRD patterns of PEA_2_SnI_4_ films with and without molecular interlayers (TPOF1, TPOF2, and 2F‐TPOF2). b) Magnified XRD patterns ≈2*θ* = 5.5^o^. c,d) XPS spectra of PEA_2_SnI_4_ films with and without molecular interlayers (TPOF1, TPOF2, and 2F‐TPOF2): (c) Sn 3d and (d) I 3d. e) Steady‐state PL emission spectra of PEA_2_SnI_4_ films with and without molecular interlayers (TPOF1, TPOF2, and 2F‐TPOF2). 410 nm wavelength laser excitation from the top side. f) UV–vis absorbance spectra of PEA_2_SnI_4_ films with and without molecular interlayers (TPOF1, TPOF2, and 2F‐TPOF2). g) ^31^P NMR spectra of the designed molecules (TPOF1, TPOF2, and 2F‐TPOF2) with and without SnI_2_. h–j) FT‐IR spectra of the designed molecules with and without SnI_2_: (h) TPOF1, (i) TPOF2, and (j) 2F‐TPOF2.

The scanning electron microscopy (SEM) image in Figure 1g confirms that a uniform surface morphology without pinholes is obtained from the pristine PEA_2_SnI_4_ thin film. This film also exhibits a highly ordered layer‐by‐layer structure with an interlayer distance (d‐spacing value) of ≈16 Å, as confirmed by X‐ray diffraction (XRD) peaks (**Figure** **2**a,b). An intense peak at 5.5° corresponding to the (002) reflection, followed by a series of weak peaks, originating from the reflections of the (00*l*) facets (*l* = 2, 4, 6, 8, 10, 12, 14), is observed. Furthermore, as shown in the UV–vis absorbance spectra (Figure 2f), the pristine PEA_2_SnI_4_ film shows three peaks at 425, 530, and 619 nm, which can be interpreted as high‐energy exciton transitions, intra‐band transition processes, and intrinsic band‐edge excitons in the crystal lattice, respectively.^[^ ^51^ ^]^

The XRD patterns and UV–vis spectra of the PEA_2_SnI_4_ films with additional interlayers on their surfaces show no peak shifts and new peak generation compared to the pristine PEA_2_SnI_4_ films (Figure 2a,b,f), suggesting that the interlayers neither alter the lattice structure nor induce a new phase in the perovskite film. Their SEM images (Figure 1h) also do not exhibit noticeable differences. Instead, a slightly enhanced intensity of XRD (Figure 2b) is observed, indicating slightly improved crystallinity. Meanwhile, a significant change is observed in photoluminescence (PL) spectra (Figure 2e), which represent non‐radiative recombination loss from the perovskite films. All films, with and without interlayers, exhibit band‐to‐band radiative PL emissions at a peak of ≈629 nm (with 410 nm excitation, irradiated from the top side where the interlayers are added), but the perovskite films with interlayers show clearly enhanced PL intensity. In particular, the TPOF1, TPOF2, and 2F‐TPOF2‐casted perovskite films presented gradually increased peak intensities, demonstrating a strong passivation effect of 2F‐TPOF2 compared to TPOF1 and TPOF2, in the following sequence: 2F‐TPOF2 > TPOF2 > TPOF1. The 2F‐TPOF2 molecule, exhibiting the strongest electron density distribution around the P═O unit (as demonstrated by molecular electrostatic potential calculations in Figure 1d–f), proves to be the most effective Lewis base passivator.^[^ ^52^, ^53^ ^]^ The passivation effect of the interlayer is further confirmed by PL spectra excited from the bottom side of the perovskite films. Given that the penetration depth of PL is limited to a few nanometers, the PL intensity excited from the bottom side–where the interlayer is not present–remains consistent regardless of the presence of the interlayer (Figure S21, Supporting Information). As the interlayer is added to the perovskite film via spin‐casting, the molecules are anticipated to more effectively address defects on the surface and within the grain boundaries, rather than influencing the overall crystallinity.

**Figure 3:**
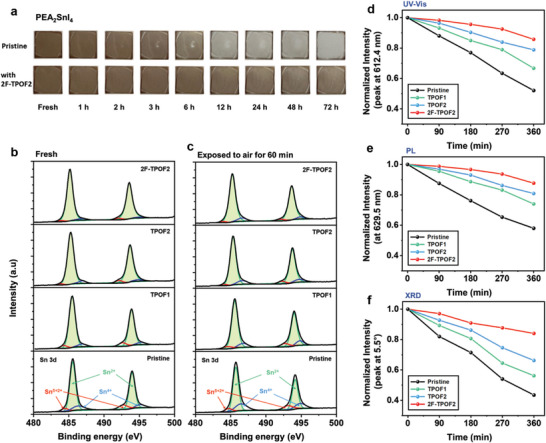
a) Photographs of the PEA_2_SnI_4_ films on a glass substrate with and without 2F‐TPOF2, representing changes in appearance over time in an atmospheric environment. b,c) High‐resolution XPS spectra of Sn 3d core levels for PEA_2_SnI_4_ films with and without molecular interlayers (TPOF1, TPOF2, and 2F‐TPOF2): (b) in the fresh state and (c) after 60 min of air exposure. d–f) Variations in normalized intensities (relative to the fresh state) extracted from the spectra in Figures S24‐S26 (Supporting Information) for different air exposure times. Black, green, blue, and red colors represent pristine PEA_2_SnI_4_, PEA_2_SnI_4_ with TPOF1, PEA_2_SnI_4_ with TPOF2, and PEA_2_SnI_4_ with 2F‐TPOF2, respectively: (d) UV–vis spectra, from absorption peak at 612.4 nm, (e) PL spectra, from emission peak at 629.5 nm, and (f) XRD pattern, from main peak at 5.5°. (a–f) All prepared samples were stored and maintained at air‐conditioned room temperature with a relative humidity of 35–45%.

To better understand the passivation of defects in the perovskite, X‐ray photoelectron spectroscopy (XPS) measurements were conducted to clarify the interaction of the designed organic molecules with the perovskite.^[^ ^54^ ^]^ As illustrated in Figure 2c and **3**b, the pristine perovskite films exhibit core‐level spectra of Sn 3d_3/2_ and Sn 3d_5/2_ with binding energies ≈494 and 485.5 eV, respectively. These binding energies slightly shift to lower values upon surface passivation (Figure 2c), with the 2F‐TPOF2 treatment presenting stronger shifts compared to TPOF1 and TPOF2. This shift is attributed to the reduction of Sn^4+^, which has higher binding energy. After deconvolution of these valence‐state peaks, the high‐resolution Sn 3d_3/2_ and Sn 3d_5/2_ spectra display two main peaks corresponding to the Sn^2+^ to Sn^4+^ states (Figure 3b), respectively.^[^ ^55^, ^56^ ^]^ The Sn^4+^ oxidation state, characterized by higher binding energy than Sn^2+^, is significantly suppressed in both Sn 3d_3/2_ and Sn 3d_5/2_ peaks, with the Sn^2+^:Sn^4+^ ratio estimated to be 86:7 in the TPOF1‐passivated film, 91:5 in the TPOF2‐passivated film, and 93:3 in the 2F‐TPOF2‐passivated film, compared to 78:12 in the pristine PEA_2_SnI_4_ film (Figure 3b). This demonstrates that Sn^4+^ generation is suppressed by the passivation of interlayers, with the effect maximized in the 2F‐TPOF2 treatment. Additionally, shoulder peaks at lower binding energies, attributed to undercoordinated Sn in the oxidation state (depicted as Sn^δ<2+^),^[^ ^54^, ^57^ ^]^ are observed with a content of 10.2% in the pristine film. These peaks almost disappear after passivation, with the content reduced to 7.7% in the TPOF1‐passivated film, 4.2% in the TPOF2‐passivated film, and 3.7% in the 2F‐TPOF2‐passivated film.

XPS measurements can also be employed to identify the presence of interlayers on the perovskite film surface. A fluorine (F) 1s signal was observed in the interlayer‐passivated PEA_2_SnI_4_ films, confirming the presence of F atoms on the perovskite film surface, whereas no distinct peak was detected in the pristine film (Figure S22, Supporting Information). The improved quality of the perovskite film, along with these bulky hydrophobic passivators, is expected to enhance the ambient stability of the PEA_2_SnI_4_ film, which will be discussed in the following section.

The chemical coordination of P═O functional groups in the designed molecules with the uncoordinated cations in the Sn perovskite was elucidated using nuclear magnetic resonance (NMR) spectroscopy (Figure 2g) and Fourier transform infrared (FT‐IR) spectroscopy (Figure 2h–j). The ^31^P NMR spectra of these molecules show significant downfield chemical shifts upon mixing with the perovskite precursor (SnI_2_), indicating changes in the electron density around the phosphorous nucleus. This observation suggests that the P═O functional groups, functioning as Lewis bases, can donate electrons to Sn^2+^, confirming their capability to interact with cationic defects in the perovskite. Furthermore, the infrared stretching vibration peak of the P═O bond (≈1194 cm^−1^), observed in the FT‐IR spectra of the designed molecules, shifts toward a lower wavenumber after mixing with the perovskite precursor component (SnI_2_), reinforcing the notion that P═O functional groups interact with Sn^2+^ cations. Especially, the relatively larger peak shifts observed for 2F‐TPOF2 in both NMR and FT‐IR spectra, compared to those for TPOF1 and TPOF2, imply a stronger interaction between P═O and Sn^2+^, in the following sequence: 2F‐TPOF2 > TPOF2 > TPOF1, which correlates with previous characterization results.

### 2.3 Enhanced Ambient Stability of PEA_2_SnI_4_ Film with Molecular Interlayer

Despite the large organic cations in 2D perovskites being bulky and hydrophobic, which act as protective barriers against environmental moisture and oxygen,^[^ ^58^ ^]^ Sn‐based 2D PEA_2_SnI_4_ perovskites are not fully insusceptible to degradation and oxidation in ambient air. The oxygen (O_2_) and hydrogen (H_2_) molecules adsorbed on the surface of perovskites can easily be converted into O_2_^−^ and hydrogen bonds, which can lead to oxidation and decomposition of Sn‐based perovskites by hydrolyzing the PEA_2_SnI_4_ to PEAI and SnI_4_.^[^ ^59^ ^]^ Considering the hydrophobic properties of the designed molecules, it is anticipated that they can effectively retard moisture intrusion into the perovskite films. The enhanced hydrophobicity of the perovskite films with the interlayers is validated by water contact angle measurements, as depicted in Figure S23 (Supporting Information). The contact angle of pristine PEA_2_SnI_4_, initially 21.7°, increased significantly to 30.3° (TPOF1), 41.2° (TPPOF2), and 46.4° (2F‐TPOF2), indicating improved ambient stability of the PEA_2_SnI_4_ film.^[^ ^60^ ^]^ As shown in **Figure** **3**a, while the PEA_2_SnI_4_ films, both with and without a passivator, exhibit a similar reddish–brown color, the PEA_2_SnI_4_ films with a passivation layer (e.g., 2F‐TPOF2) demonstrate mitigated degradation compared to the pristine PEA_2_SnI_4_ in the atmospheric environment over a period of 72 h. This observation indicates superior stability of the passivated PEA_2_SnI_4_ film.

To further evaluate the stability of the PEA_2_SnI_4_ films with and without surface passivation, we analyzed the variation in UV–vis and PL spectra of the PEA_2_SnI_4_ films depending on air‐exposure time. Figure 3d,e reveal the optical properties (UV–vis absorbance and PL, respectively) of the PEA_2_SnI_4_ films with and without passivators at different air exposure times. The intensities of peaks, extracted from each main peak (at 612.4 and 629.5 nm, respectively), were normalized relative to their intensities in the fresh state to better monitor changes. For reference, the UV–vis and PL spectra are provided in Figures S24 and S25 (Supporting Information). Clearly, the pristine PEA_2_SnI_4_ film exhibits rapid degradation with increasing air exposure time, with the respective peak intensities of UV–vis and PL spectra decaying to nearly half their values in the fresh state after 360 min. In contrast, the PEA_2_SnI_4_ films with the passivators retained ≈70–90% of the initial peak intensities, representing higher ambient stability compared to the pristine film. Particularly, the stability of the PEA_2_SnI_4_ film with 2F‐TPOF2, having the highest number of F groups, is the highest.

We also tracked the changes of the intense main peak at 5.5° in the XRD patterns of the PEA_2_SnI_4_ films, with and without the surface passivation, for different air exposure times (Figure S26, Supporting Information). The peak intensity at 5.5° continued to decrease with air exposure time for all films but at different rates. Figure 3f displays the normalized peak intensity at 5.5° relative to the fresh state for different air exposure times. The peak intensity of the pristine PEA_2_SnI_4_ film at 5.5° started to decline dramatically after 90 min of air exposure and decayed to less than half its initial value after 360 min. In contrast, the PEA_2_SnI_4_ films with passivators retained more than 60% of the initial peak intensity even after 360 min, confirming the ability of the surface passivation layer to retard moisture intrusion. The effectiveness of ambient stability improvement follows the sequence: 2F‐TPOF2 > TPOF2 > TPOF1, corresponding to the number of fluorine functional groups in each molecule. Additionally, the degradation of Sn‐perovskite can be also accessed by the increased ratio of Sn^4+^ to Sn^2+^.^[^ ^61^ ^]^ The variation of Sn^4+^ composition can be evaluated by the XPS spectra (Figure 3b,c), and we confirm that the content of Sn^4+^ and Sn^δ<2+^ species in the films exposed to air for 60 min is suppressed with the passivators, with the effect maximized in the 2F‐TPOF2 treatment.

### 2.4 Energy Barrier Reduction Between Au and PEA_2_SnI_4_ Film with Molecular Interlayer

**Figure 4:**
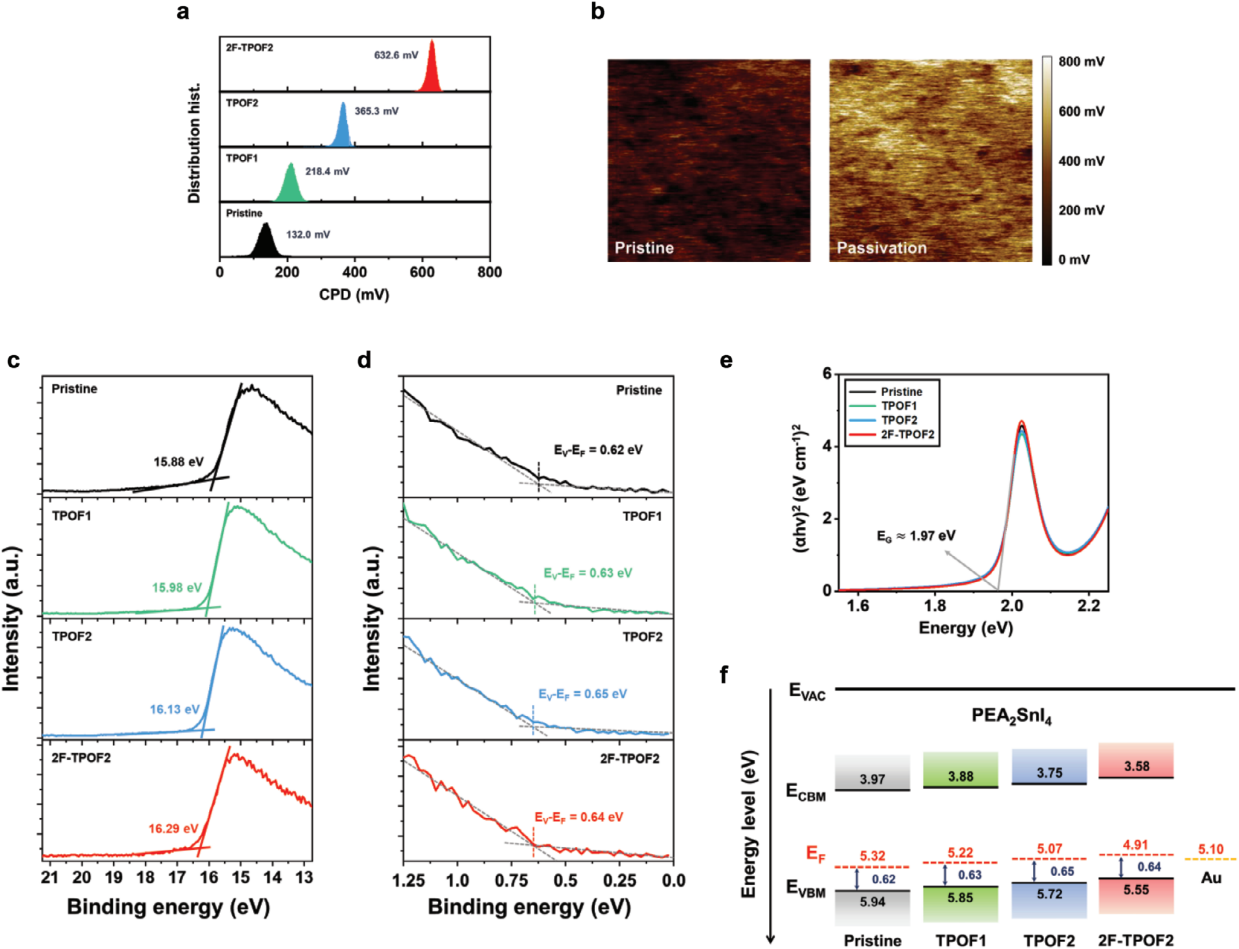
a) Measured surface potentials of PEA_2_SnI_4_ films on Si/SiO_2_ substrates with and without molecular interlayers (TPOF1, TPOF2, and 2F‐TPOF2). Hybrid film‐tip distance for surface potential measurements is 75 nm for all conditions (resolution: 256 pixels × 256 pixels). b) 2D surface potential maps of pristine PEA_2_SnI_4_ film and PEA_2_SnI_4_ film with 2F‐TPOF2. c,d) UPS binding energy profiles of PEA_2_SnI_4_ films with and without molecular interlayers (TPOF1, TPOF2, and 2F‐TPOF2): (c) secondary electron cut‐off energy region, indicating work function, and (d) valence band region. e) Tauc plots showing the optical bandgap energy (*E*_G_) of PEA_2_SnI_4_ films with and without molecular interlayers (TPOF1, TPOF2, and 2F‐TPOF2). f) Energy band diagrams of PEA_2_SnI_4_ films with and without molecular interlayers (TPOF1, TPOF2, and 2F‐TPOF2), representing VBM, CBM, and Fermi energy levels.

The strong dipole moments of the designed molecules, inserted between the PEA_2_SnI_4_ film and Au source/drain, affect the energy barrier at their interface. To understand this dipole moment effect, we characterize the variation in the work function of the perovskite films using Kelvin probe force microscope (KPFM) analyses^[^ ^62^, ^63^ ^]^ (**Figure** **4**a,b). As previously confirmed, the P═O unit, exhibiting the highest electron density distribution (Figure 1d–f) and contributing to a negative dipole, efficiently coordinates with the perovskite. Concurrently, the other side of the molecule, where a positive dipole is dominant, is oriented toward the Au source/drain. This configuration is expected to decrease the work function of perovskite, facilitating the formation of ohmic contact with the Au source/drain. The contact potential difference (CPD), defined as the difference between the work function of the tip and that of the sample surface,^[^ ^64^ ^]^ increases sharply after surface passivation with the molecules. The average CPD values of the surface‐passivated PEA_2_SnI_4_ films (218.4, 365.3, and 632.6 mV for TPOF1, TPOF2, 2F‐TPOF2, respectively) are significantly higher than that of the pristine PEA_2_SnI_4_ film (132.0 mV). Since a higher CPD value indicates a sample with a lower work function, this suggests that the work function of perovskite decreases with the interlayer and this effect is most pronounced in 2F‐TPOF2 and least in TPOF1, correlating with the strength of the dipole moment,^[^ ^65^ ^]^ as expected.

The work function variation of the perovskite with passivation was further evaluated using UV photoelectron spectroscopy (UPS) analysis.^[^ ^66^ ^]^ From the secondary electron cut‐off energy (*E*_cut − off_) (Figure 4c), the work function (φ) is calculated: φ  =  *h*ν − |*E*_cut − off_ − *E*_F_|, and, from the onset energy (Figure 4d), the valence band maximum (VBM) is estimated. Additionally, the conduction band minimum is estimated from the optical bandgap of each film via its Tauc plot (Figure 4e), derived from UV–vis absorbance spectra. As observed earlier, all prepared films exhibit a constant band gap value of ≈1.97 eV. The estimated energy band diagrams of the pristine and passivated PEA_2_SnI_4_ films are summarized in Figure 4f. Consistent with the KPFM measurement results, the work function of PEA_2_SnI_4_ decreases with passivation–raising the Fermi energy level along with the VBM–and is lowest with 2F‐TPOF2, which has the strongest dipole moment. For reference, the difference between the Fermi energy level and VBM of the perovskite with 2F‐TPOF2 (0.64 eV) exhibits a slight increase compared to the pristine perovskite (0.62 eV), indicating a reduction in the p‐doping level of the perovskite. The p‐doping is often attributed to the Sn vacancies (*V*_Sn_), formed through the oxidation of Sn^2+^ to Sn^4+^, which create shallow acceptor levels associated with *V*_Sn_.^[^ ^57^, ^67^, ^68^, ^69^ ^]^ These vacancies can disrupt the uniformity of the perovskite crystal lattice, introducing defects that localize charge carriers in trap states. This hinders the complete reversibility of the system and contributes to significant degradation over time.^[^ ^70^ ^]^

### 2.5 Performance of PEA_2_SnI_4_ FETs

**Figure 5:**
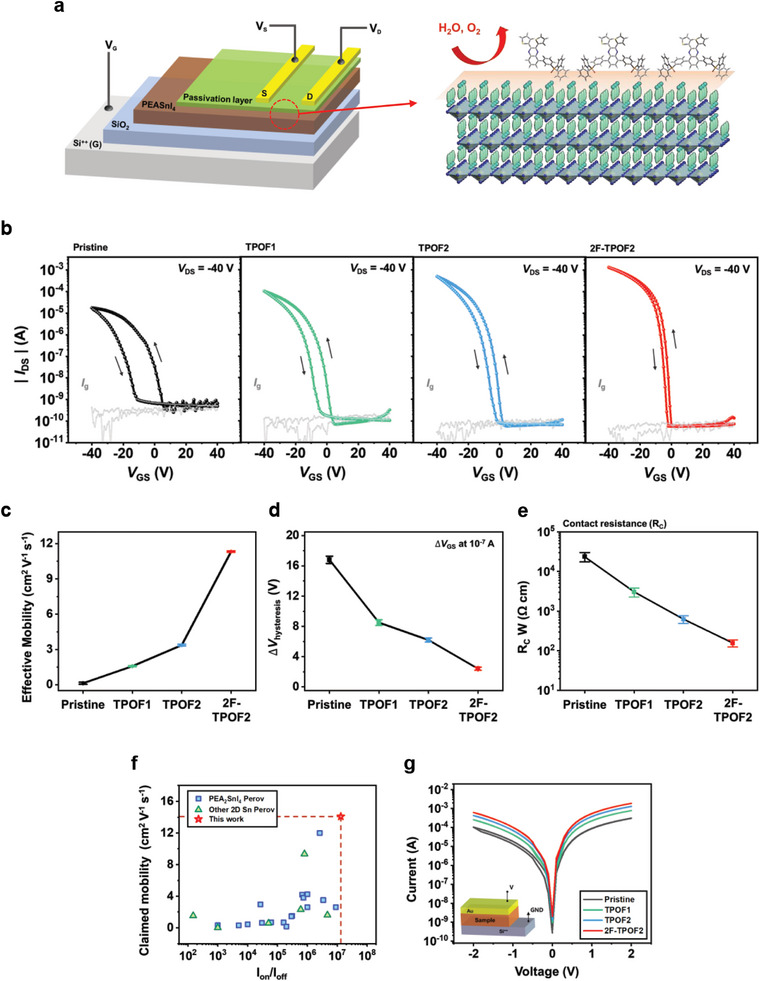
a) 3D schematic illustration of a PEA_2_SnI_4_ perovskite FET with molecular interlayer passivation in a bottom‐gate top‐contact configuration. b) Transfer characteristics of FETs fabricated by pristine PEA_2_SnI_4_ and PEA_2_SnI_4_ with molecular interlayers (TPOF1, TPOF2, and 2F‐TPOF2). Measurements were taken at *V*_DS_ = ‐40 V; grey lines denote gate leakage currents. Channel width and length are 1500 and 100 µm. Transfer curves were obtained at a scan rate of 2 V s^−1^. c–e) Statistical analyses of PEA_2_SnI_4_ FETs with and without molecular interlayers (TPOF1, TPOF2, and 2F‐TPOF2). Error bars represent data from 30 devices per type, presented as mean ± standard deviation: (c) effective average hole mobility, (d) hysteresis, and (e) contact resistance. f) Mobility versus *I*_on_/*I*_off_ values of 2D Sn halide perovskite FETs in literature (data in Table S3, Supporting Information). g) Current–voltage curves under dual sweep for device sandwiching PEA_2_SnI_4_, with and without molecular interlayers (TPOF1, TPOF2, and 2F‐TPOF2), between p^+^‐Si and Au electrode, evaluating out‐of‐plan transport. The inset shows the device structure.

The impact of surface passivation and ohmic contact enhancement on the performance of the PEA_2_SnI_4_ FET devices is systematically investigated. **Figure** **5**a depicts the architecture of the FET device, which incorporates a surface‐passivated PEA_2_SnI_4_ channel layer grown on a p^+^‐Si/silicon dioxide (SiO_2_, 300 nm) substrate. A bottom‐gate top‐contact configuration with a channel length of 100 µm and a width of 1500 µm was adopted for device fabrication. Detailed fabrication procedures are provided in the Experimental Section. The electrical characteristics were measured under ambient air conditions at room temperature, following glass encapsulation.

The incorporation of the interlayers significantly enhances the FET device performance, with the extent of improvement correlating closely with the dipole moment strength, which determines the effectiveness of defect‐passivation on the perovskite and the reduction of the interfacial energy barrier with the Au source/drain. Figure 5b presents the transfer curves (at *V*_D_ = ‐40 V in the saturation regime) for devices with and without passivators (TPOF1, TPOF2, and 2F‐TPOF2), and the corresponding output curves are shown in Figure S27 (Supporting Information).^[^ ^71^ ^]^ The surface‐passivated FET devices exhibit markedly improved performance compared to the pristine device. Especially, the 2F‐TPOF2 FETs achieve the highest performance, with a mobility (µ_claimed_) of 14.02 cm^2^ V^−1^ s^−1^, an on/off current ratio (*I*_on/off_) of 1.32 × 10^7^, a turn‐on voltage (*V*_on_) of 1.1 V, and a subthreshold swing (SS) of 0.89 V dec^−1^. For reference, the performance variation of FETs depending on the thickness of 2F‐TPOF2 is investigated in Figure S28 (Supporting Information), with 0.25 mg mL^−1^ displaying the best performance. The performance metrics of 2F‐TPOF2 FETs represent significant enhancements over the pristine FET, which display a µ_claimed_ of 0.27 cm^2^ V^−1^ s^−1^, an *I*_on/off_ of 1.12  × 10^5^, a *V*_on_ of 8.7 V, and an SS of 2.84 V dec^−1^. All these parameters are average values derived from 30 different devices, and the statistical data, including the performance metrics for FETs utilizing other molecules such as TPOF1 and TPOF2, are summarized in Table S2 (Supporting Information). Table S3 (Supporting Information) and Figure 5f demonstrate that the performance of FETs with 2F‐TPOF2 surpasses other 2D Sn perovskite‐based FET devices reported in the literature. For reference, the aforementioned parameters are derived from the transfer curves scanned in the forward direction (off‐to‐on). To secure the reliability of the mobility values, a measurement reliability factor (*r*), used to precisely estimate mobility, is estimated, and its value and the effective mobility (µ_effective_ = *r* × µ_claimed_) are summarized in Figure 5c and Table S4 (Supporting Information). For reference, Figure 5c represents the effective mobility statics (mean and standard deviation) from 30 FET devices, each fabricated with and without various molecular interlayers. The mean (standard deviation) of µ_effective_ for these devices is 1.58 cm^2^ V^−1^ s^−1^ (0.044) for TPOF1, 3.37 cm^2^ V^−1^ s^−1^ (0.035) for TPOF2, and 11.32 cm^2^ V^−1^ s^−1^ (0.031) for 2F‐TPOF2, compared to 0.13 cm^2^ V^−1^ s^−1^ (0.052) for the pristine condition. Detailed methods for calculating *r* values and extracting reliable mobility values are explained in Note S1 (Supporting Information).

Meanwhile, the pristine FET exhibits significant dual‐sweep hysteresis, as depicted in Figure S29a (Supporting Information). It has been reported that I interstitials/vacancies (Frenkel defects), which are preferentially formed under Sn vacancy‐rich conditions, are principal defects inducing hysteresis.^[^ ^57^ ^]^ In the I 3d XPS spectra (Figure 2d), shoulder peaks appear at higher binding energy in the pristine thin film, corresponding to the I_3_^−^ species, indicative of I interstitials/vacancies. These Frenkel defects are significantly reduced with the passivation interlayers and are almost entirely suppressed with the 2F‐TPOF2. Therefore, the passivation of Sn perovskite with these interlayers is expected to mitigate hysteresis in FET devices. Figure S29b–d (Supporting Information) shows a substantial reduction in hysteresis in FET devices with passivators, with the hysteresis nearly eliminated in the FET incorporating 2F‐TPOF2. Figure 5d presents hysteresis statics from 30 different devices (the width of the hysteresis for *V*_GS_ (▵*V*) is calculated at. |*I*_DS_| = 10^−7^ A in both sweep directions, approximately halfway between the on and off states).^[^ ^57^, ^72^ ^]^ The devices with passivators exhibit a reduced mean (standard deviation) ▵*V* of 8.5 V (0.381) for TPOF1, 6.2 V (0.272) for TPOF2, and 2.4 V (0.214) for 2F‐TPOF2, compared to 16.8 V (0.486) for the pristine PEA_2_SnI_4_ device.

The effect of energy barrier reduction between the perovskite and Au source/drain on the device performance is assessed by evaluating the contact resistance (*R*_c_) at their interface. *R*_c_ values were calculated using the transmission line method,^[^ ^73^ ^]^ and their variations depending on the passivator, based on data from 30 different devices for each condition, are summarized in Figure 5e. The mean (standard deviation) *R*_c_ for the pristine devices is 2.37 × 10^4^ Ω cm (0.633), reduced to 3.13 × 10^3^ Ω cm (0.512) for TPOF1, 6.44 × 10^2^ Ω cm (0.143) for TPOF2, and 1.61 × 10^2^ Ω cm (0.034) for 2F‐TPOF2 FETs. Detailed plots of total resistance as a function of channel length are presented in Figure S30 (Supporting Information). Additionally, this enhanced charge transport through the interface between the perovskite and Au source/drain is further evaluated by comparing the out‐of‐plan charge transport capabilities of two‐terminal devices, prepared by sandwiching PEA_2_SnI_4_ between p^+^‐Si and Au electrode (inset of Figure 5g). As shown in Figure 5g, the devices with the passivated PEA_2_SnI_4_ exhibit much higher current than those with the pristine PEA_2_SnI_4_, with the 2F‐TPOF2 device demonstrating the most efficient out‐of‐plane charge transport from the perovskite to the Au electrode without hysteresis.

### 2.6 Operational Stability of PEA_2_SnI_4_ FETs

**Figure 6:**
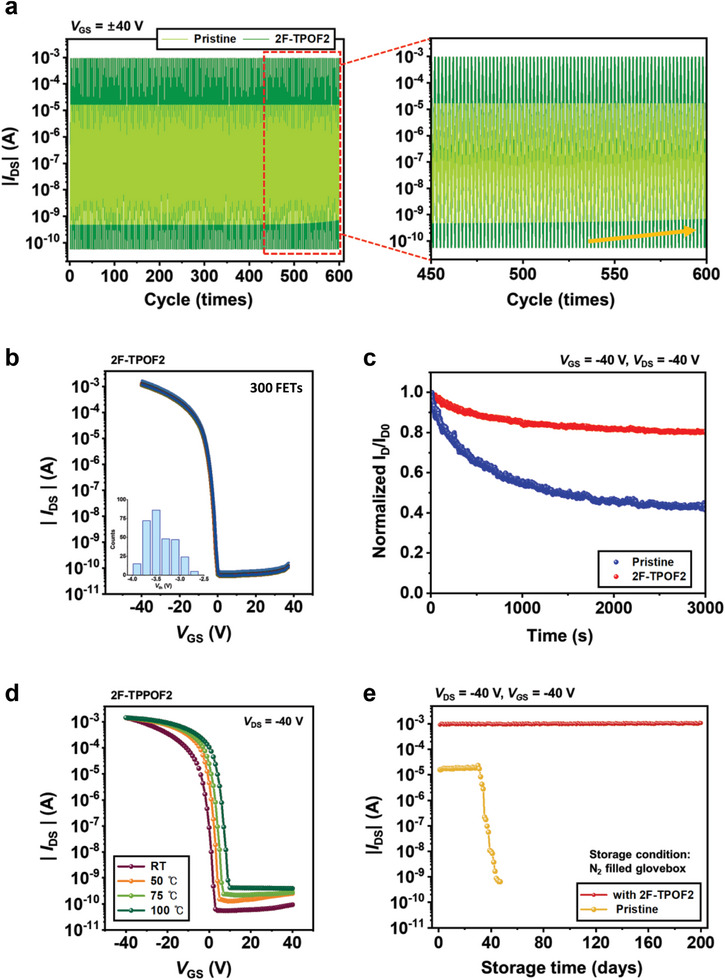
a) Continuous on/off switching characteristics of PEA_2_SnI_4_ FETs with and without 2F‐TPOF2 during 600 cycles. Measurement conditions: *V*_GS_ = ± 40 V; the pulse condition involves a 40 V turn‐on pulse for 1 s followed by a ‐40 V turn‐off pulse for 1 s. b) Transfer curves for 300 different 2F‐TPOF2‐passivated PEA_2_SnI_4_ FETs fabricated from 20 different batches (*V*_DS_ = ‐40 V). The inset figure presents the threshold voltage distribution of the 300 FETs. c) Bias‐stress stability of PEA_2_SnI_4_ FETs with and without 2F‐TPOF2 for over 3000 s under constant bias conditions (*V*_DS_ = ‐40 V and *V*_GS_ = ‐40 V). d) Temperature‐dependent transfer characteristics of PEA_2_SnI_4_ FETs with and without 2F‐TPOF2 at various temperatures up to 100 °C (*V*_DS_ = ‐40 V). e) Storage stability of PEA_2_SnI_4_ FETs with and without 2F‐TPOF2 as a function of storage time in an N_2_ glovebox over 200 days (*V*_DS_ = ‐40 V and *V*_GS_ = ‐40 V).

As previously discussed, the surface passivation of perovskite films with the designed molecules enhances their film quality, leading to improved ambient stability. In this context, the operational stability of the FET devices, a critical factor for their development and practical applications,^[^ ^74^ ^]^ is anticipated to be enhanced. **Figure** **6**a presents the on/off switching stability of the devices with and without surface passivation, with the 2F‐TPOF2‐treated device selected as a representative of the surface‐passivated devices. The pristine device exhibits an increase in off‐state current after 450 cycles, whereas the optimized 2F‐TPOF2‐treated device maintains consistent current response in both the on and off states over 600 cycles, indicating more reliable operation of the surface‐passivated devices. Additionally, as demonstrated in Figure 6b, the 2F‐TPOF2‐treated TFTs show high reproducibility. A histogram from 300 devices across twenty batches (inset of Figure 6b) reveals a narrow distribution of *V*_T_, with an average of 3.42 V and a standard deviation of 0.29 V, confirming the consistency of the devices.

To rigorously assess the operational stability of the FET devices, we performed negative‐bias stress (NBS) measurements, monitoring the output current (*I*_DS_) under a constant negative gate and drain, voltages (*V*_GS_ = ‐40 V, and *V*_DS_ = ‐40 V). As depicted in Figure 6c, the *I*_DS_ of the pristine device rapidly decreases to less than half of its initial value after only 1000 s, reflecting significant carrier trapping within the device. In contrast, the defect‐passivated 2F‐TPOF2 device exhibits substantially enhanced stability, with minimal *I*_DS_ decay of less than 15% from its initial value even after 3000 s, primarily due to the reduced trap states.^[^ ^57^ ^]^

To evaluate thermal stability, we monitored changes in device performance at elevated temperatures ranging from room temperature (25 °C) to 100 °C (Figure 6d). The 2F‐TPOF2 device demonstrates superior stability with slight fluctuations in the transfer curve, whereas the pristine FET devices show significant performance variation with temperature (Figure S31, Supporting Information). For reference, the ease of activating ionic defects within the lattice can result in severe ion migration in perovskite films when exposed to external stimuli, which subsequently degrades device performance.^[^ ^75^ ^]^ Therefore, the reduced defect density, and the corresponding suppression of ion migration in the 2F‐TPOF2 device, are expected to enhance its thermal stability. Furthermore, due to the hydrophobic nature of the surface passivator, enhanced storage stability is anticipated. As shown in Figure 6e, the 2F‐TPOF2 device maintains its on‐current level with negligible degradation for over 200 days, significantly outperforming the pristine device, thus providing excellent storage stability for an optimized 2F‐TPOF2 device (in an N_2_‐filled glove box with glass encapsulation).

## 3 Conclusion

We develop quinoxaline‐phosphine oxide‐based small molecules (TPOF1, TPOF2, and 2F‐TPOF2) designed to passivate surface defects in 2D RP PEA_2_SnI_4_ perovskite and to facilitate the formation of ohmic contacts at the source and drain interfaces. From a passivation standpoint, the P═O groups, modified with fluorine atoms, enable these molecules to form Lewis adducts with halogen vacancies in the Sn perovskites, thereby reducing the surface defects and preventing the oxidation of Sn^2+^–Sn^4+^. Among the synthesized molecules, 2F‐TPOF2, featuring an enhanced dipole moment due to strategic fluorine positioning, exhibits the highest efficacy. The passivation effectiveness is validated by XPS and PL analyses, which reveal a substantial reduction in surface defects and suppressed non‐radiative recombination in the treated films. The hydrophobic nature of the fluorine groups also enhances the moisture resistance, contributing to improved ambient stability of the films. In terms of energy‐barrier adjustment, the strong molecular dipole moment of 2F‐TPOF2 effectively lowers the work function of the perovskite films, as confirmed by KPFM and UPS analyses. This adjustment promotes the formation of ohmic contacts with the Au source and drain electrodes. Consequently, the 2F‐TPOF2‐passivated PEA_2_SnI_4_ FETs exhibit significantly improved performance metrics, inducing carrier mobility of 14.08 cm^2^V⁻¹s⁻¹ and *I*_on/off_ ratio of 1.32 × 10^7^, alongside a marked reduction in hysteresis. Furthermore, these FET devices demonstrate exceptional durability, and reproducibility, as well as superior thermal and storage stability. These advancements emphasize the potential of molecular engineering in enhancing both the charge transport properties and stability of Sn‐based perovskite FETs, highlighting a promising avenue for the development of high‐performance perovskite electronics.

## Conflict of Interest

The authors declare no conflict of interest.

## Supporting information



Supporting Information

## Data Availability

The data that support the findings of this study are available from the corresponding author upon reasonable request.
