# Supplementary material for: Molecular Interlayer for High‐Performance and Stable 2D Tin Halide Perovskite Transistor
Source: Adv Sci (Weinh). 2025 Apr 9;12(26):2409088. doi: 10.1002/advs.202409088 (PMC12245131; doi:10.1002/advs.202409088)
Supplement: Supplementary file 1 — Supporting Information [file ADVS-12-2409088-s001.pdf]

ADVANCED SCIENCE
Open Access

Supporting Information

for *Adv. Sci.*, DOI 10.1002/advs.202409088

Molecular Interlayer for High-Performance and Stable 2D Tin Halide Perovskite Transistor

Bum Ho Jeong, Juan Anthony Prayogo, Jongmin Lee, Seok Woo Lee, Dong Ryeol Whang, Dong Wook Chang and Hui Joon Park**

Supporting Information

Molecular interlayer for high-performance and stable 2D tin halide perovskite transistor

Bum Ho Jeong, Juan Anthony Prayogo, Jongmin Lee, Seok Woo Lee, Dong Ryeol Whang, Dong Wook Chang, Hui Joon Park**

B. H. Jeong, J. Lee, Prof. H. J. Park
Department of Organic and Nano Engineering & Human-Tech Convergence Program, Hanyang University, Seoul 04763, Republic of Korea
E-mail: huijoon@hanyang.ac.kr

J. A. Prayogo, S. W. Lee, Prof. D. W. Chang
Department of Industrial Chemistry and CECS Research Institute, Pukyong University, Busan 48513, Republic of Korea
E-mail: dwchang@pknu.ac.kr

Prof. D. R. Whang
Division of Advanced Materials, Hannam University, Daejeon 34054, Republic of Korea

Prof. H. J. Park
Department of Semiconductor Engineering, Hanyang University, Seoul 04763, Republic of Korea

Experimental Section

General synthetic procedure: Bis(4-fluorophenyl)(phenyl)phosphine oxide (**1**), 4-bromobenzo[c][1,2,5]thiadiazole (**4**), 4,7-Dibromobenzo[c][1,2,5]thiadiazole (**5**), 4,7-Dibromo-5,6-difluorobenzo[c][1,2,5]thiadiazole (**6**), and 5,8-dibromo-2,3-di(thiophen-2-yl)quinoxaline (**8**) in Figure S1 (Supporting Information) were prepared according to previously reported literatures.[1-4] For reference, 2,2'-Thenil and all other reagents and solvents were purchased from Sigma Aldrich and TCI Chemicals.

(3-Bromophenyl)bis(4-fluorophenyl)phosphine oxide (**2** in Figure S1 ,Supporting Information): In a round-bottomed flask, bis(4-fluorophenyl)(phenyl)phosphine oxide (**1** in Figure S1 (Supporting Information), 1eq., 10 mmol) was stirred in 30 ml of 95% sulfuric acid until **1** in Figure S1 (Supporting Information) was completely dissolved. After adding *N*-bromosuccinimide (NBS, 1.1 eq., 11 mmol)**,** the mixture was stirred for 4 days at room temperature. Upon completion of the reaction, the reaction mixture was poured into ice water and extracted with dichloromethane three times. The organic layers were separated, dried over magnesium sulfate, and filtered. The solvent was removed under reduced pressure, and the crude residue was purified by column chromatography using ethyl acetate/dichloromethane (1/1, v/v) as an eluent. Yield =68% (White solid). ^{1}H NMR (400 MHz, $CDCl_3$) δ (ppm) = 7.77 (dt, J = 12.2, 1.7 Hz, 1H), 7.60-7.69 (m, 5H), 7.51-7.56 (m, 1H), 7.35 (td, J = 7.9, 3.5 Hz, 1H), 7.18 (td, J = 8.7, 2.1 Hz, 4H). ^{13}C NMR (100 MHz, $CDCl_3$): δ (ppm) = 166.66, 166.63, 164.13, 164.10, 135.46, 135.44, 134.72, 134.68, 134.62, 134.60, 134.51, 132.06, 131.96, 130.52, 130.48, 130.43, 130.36, 129.00, 128.87, 128.23, 128.19, 127.15, 127.12, 123.53, 123.37, 116.51, 116.38, 116.30, 116.17. ^{19}F NMR (376 MHz, $CDCl_3$): δ (ppm) = -105.45. ^{31}P NMR (162 MHz, $CDCl_3$): δ (ppm) = 27.05.

Bis(4-fluorophenyl)(3-(4,4,5,5-tetramethyl-1,3,2-dioxaborolan-2-yl)phenyl)phosphine oxide (**3** in Figure S1, Supporting Information): A mixture of **2** in Figure S1 (Supporting Information) (1 eq., 11.7 mmol), bis(pinacolato)diboron (1.2 eq., 14.04 mmol), potassium acetate (3.5 eq., 40.9 mmol), and [1,1'-bis(diphenylphosphino)ferrocene]dichloropalladium(II) (Pd(dppf)Cl_2, 5 mol%) in dry toluene (50 ml) was stirred at 100 °C for 24 h under N_2 atmosphere. Upon completion of the reaction, the mixture was poured into water and extracted with dichloromethane. The organic solvent layer was separated from water and dried over magnesium sulfate. The crude product was purified by column chromatography on a silica gel using ethyl acetate/chloroform (1/4, v/v) as an eluent. Yield = 32% (White solid). ^{1}H NMR (400 MHz, $CDCl_3$) δ (ppm) = 8.17 (d, J = 11.9 Hz, 1H), 7.96 (dd, J = 7.3, 1.4 Hz, 1H), 7.59-7.66 (m, 5H), 7.44 (td, J = 7.5, 3.0 Hz, 1H), 7.11-7.16 (m, 4H), 1.29 (s, 12H). ^{13}C NMR (100 MHz, $CDCl_3$): δ (ppm) 166.47, 166.44, 163.95, 163.92, 138.60, 138.57, 138.22, 138.12, 134.75, 134.66, 134.64, 134.56, 134.46, 131.88, 130.84, 129.10, 129.06, 127.97, 127.86, 116.26, 116.13, 116.05, 115.92, 84.27, 24.94. ^{19}F NMR (376 MHz, $CDCl_3$): δ (ppm) = -106.40. ^{31}P NMR (162 MHz, $CDCl_3$): δ (ppm) = 28.61.

5-Bromo-2,3-di(thiophen-2-yl)quinoxaline (**7** in Figure S1, Supporting Information): In a round-bottomed flask, **4** in Figure S1 (Supporting Information) (1 eq., 2.32 mmol) and zinc powder (18 eq., 42 mmol) were stirred in acetic acid (20 mL) at 85 °C for 1 h. After the reaction was completed, zinc power was removed by filtration, and the filtrate was collected. After addition 2,2'-thenil (1.2 eq., 2.78 mmol), the

mixture was heated to reflux overnight. The solution was cooled to room temperature, and the mixture was poured into water and extracted with dichloromethane. The organic layer was separated and dried over magnesium sulfate. The solvent was removed under reduced pressure, and the crude residue was purified by column chromatography using dichloromethane/hexane (1/2, v/v) as an eluent. Yield = 58% (light yellow solid). ^{1}H NMR (400 MHz, $CDCl_3$) δ (ppm) = 8.00-8.03 (m, 2H), 7.51-7.57 (m, 3H), 7.37-7.39 (m, 1H), 7.33 (dd, J = 3.9, 1.1 Hz, 1H), 7.08 (dd, J = 5.0, 3.7 Hz, 1H), 7.02 (dd, J = 5.0, 3.7 Hz, 1H). ^{13}C NMR (100 MHz, $CDCl_3$): δ (ppm) = 147.09, 146.94, 141.62, 141.15, 140.61, 138.38, 133.46, 130.09, 130.07, 129.87, 129.68, 129.24, 128.55, 127.69, 127.63, 123.64.

5,8-Dibromo-6,7-difluoro-2,3-di(thiophen-2-yl)quinoxaline (**9** in Figure S1, Supporting Information): In a round-bottomed flask, **6** in Figure S1 (Supporting Information) (1 eq., 2.30 mmol) and zinc powder (20 eq., 46 mmol) were stirred in acetic acid (30 mL) at 85 °C for 1 h. After the reaction was completed, zinc power was removed by filtration and the filtrate was collected. After adding 2,2'-thenil (1.2 eq., 2.76 mmol), the mixture was heated to reflux overnight. The solution was cooled to room temperature, and the mixture was poured into water and extracted with dichloromethane. The organic layer was separated and dried over magnesium sulfate. The solvent was removed under reduced pressure, and the crude residue was purified by column chromatography using dichloromethane/hexane (1/2, v/v) as an eluent. Yield = 65% (light yellow solid). ^{1}H NMR (400 MHz, $CDCl_3$) δ (ppm) = 7.55 (dd, J = 5.0, 0.9 Hz, 2H), 7.46 (dd, J = 3.9, 1.1 Hz, 2H), 7.04 (dd, J = 5.0, 3.7 Hz, 2H). ^{13}C NMR (100 MHz, $CDCl_3$): δ (ppm) = 152.05, 151.85, 149.48, 147.31, 140.53, 135.54, 130.74, 130.37, 127.90, 109.12, 109.04. ^{19}F NMR (376 MHz, $CDCl_3$): δ (ppm) = -119.01.

(3-(2,3-Di(thiophen-2-yl)quinoxalin-5-yl)phenyl)bis(4-fluorophenyl)phosphine oxide **(TPOF1)**: In a Schlenk flask, **7** in Figure S1 (Supporting Information) (1eq., 0.40 mmol), **3** in Figure S1 (Supporting Information) (1.2 eq., 0.48mmol), and $Pd(PPh_3)_4$ (5 mol%) were dissolved in dry toluene (9 mL), and then 2M aqueous solution of potassium carbonate (3 mL) was added. After bubbling with N_2 for 15 min, the mixture was stirred at 90 °C for 2 days under N_2 protection. Upon completion of the reaction, the reaction mixture was poured into water and extracted with dichloromethane. The organic solvent layer was separated from water and dried over anhydrous magnesium sulfate. After removal of the solvent under reduced pressure, the crude residue was purified by column chromatography on a silica gel using hexane/ethyl acetate (1/2, v/v) as an eluent. Finally, the product was collected and dried overnight in a vacuum oven at 60°C. Yield = 69% (Yellow solid). ^{1}H NMR (400 MHz, $CDCl_3$) δ (ppm) = 8.03-8.07 (m, 2H), 7.99 (dd, J = 7.1, 1.6 Hz, 1H), 7.67-7.73 (m, 7H), 7.62-7.65 (m, 1H), 7.50 (dd, J = 5.0, 1.4 Hz, 1H), 7.31 (qd, J = 4.3, 1.1 Hz, 2H), 7.08-7.15 (m, 5H), 7.04-7.05 (m, 1H), 6.91 (dd, J = 5.0, 3.7 Hz, 1H). ^{13}C NMR (100 MHz, $CDCl_3$): δ (ppm) = 166.47, 166.43, 163.95, 163.91, 146.23, 146.13, 142.34, 141.07, 140.66, 138.71, 138.68, 138.59, 138.14, 134.91, 134.82, 134.79, 134.71, 134.46, 134.35, 132.24, 131.16, 131.07, 130.72, 129.85, 129.45, 129.40, 129.05, 129.01, 128.53, 128.40, 127.93, 127.70, 116.26, 116.13, 116.05, 115.92. ^{19}F NMR (376 MHz, $CDCl_3$): δ (ppm) = -106.31. ^{31}P NMR (162 MHz, $CDCl_3$): δ (ppm) = 28.55. HR Q-TOF MS: m/z calcd, 606.0801; found, 606.0807 [M^+].

((2,3-Di(thiophen-2-yl)quinoxaline-5,8-diyl)bis(3,1-phenylene))bis(bis(4-fluorophenyl)phosphine oxide) **(TPOF2)**: The TPOF2 was synthesized in a similar procedure to that of TPOF1. The compound of **8** in Figure S1 (Supporting Information) was used as the reagent instead of **7** . The crude residue was purified by column chromatography using dichloromethane/ethyl acetate (1/1, v/v) as an eluent. Yield = 65% (yellow solid). ^{1}H NMR (400 MHz, $CDCl_3$) δ (ppm) = 8.08 (d, J = 13.3 Hz, 2H), 7.98-8.01 (m, 2H), 7.76 (s, 2H), 7.66-7.72 (m, 10H), 7.63 (t, J = 3.0 Hz, 2H), 7.31-7.33 (m, 2H), 7.27 (dd, J = 3.8, 0.8 Hz, 2H), 7.09-7.14 (m, 8H), 6.94 (dd, J = 5.0, 3.7 Hz, 2H). ^{13}C NMR (100 MHz, $CDCl_3$): δ (ppm) = 166.48, 166.45, 163.96, 163.93, 145.35, 141.91, 138.54, 138.45, 138.42, 137.94, 134.91, 134.82, 134.79, 134.71, 134.65, 134.48, 134.37, 132.31, 131.26, 131.14, 130.20, 129.48, 129.43, 129.05, 129.03, 128.57, 128.44, 127.98, 127.95, 127.67, 116.28, 116.15, 116.07, 115.94. ^{19}F NMR (376 MHz, $CDCl_3$): δ (ppm) = -106.24. ^{31}P NMR (162 MHz, $CDCl_3$): δ (ppm) = 28.55. HR Q-TOF MS: m/z calcd, 918.1317; found, 918.1320 [M].

((6,7-Difluoro-2,3-di(thiophen-2-yl)quinoxaline-5,8-diyl)bis(3,1-phenylene))bis(bis(4-fluorophenyl)phosphine oxide) **(2F-TPOF2)**: The 2F-TPOF2 was synthesized in a similar procedure to that of TPOF1. The compound of **9** in Figure S1 (Supporting Information) was used as the reagent instead of **7** in Figure S1 (Supporting Information). The crude residue was purified by column chromatography using dichloromethane/ethyl acetate (1/1, v/v) as an eluent. Yield = 62% (yellow solid). ^{1}H NMR (400 MHz, $CDCl_3$) δ (ppm) = 7.87-7.90 (m, 4H), 7.65-7.79 (m, 12H), 7.32 (dd, J = 5.0, 0.9 Hz, 2H), 7.25 (dd, J = 3.7, 0.9 Hz, 2H), 7.09-7.15 (m, 8H), 6.93 (dd, J = 5.0, 3.7 Hz, 2H). ^{13}C NMR (100 MHz, $CDCl_3$): δ (ppm) = 166.50, 166.48, 163.98, 163.96, 145.72, 141.35, 135.64, 135.60, 135.44, 135.33, 135.23, 134.89, 134.80, 134.79, 134.69, 132.51, 132.10, 132.01, 131.46, 130.85, 130.71, 129.80, 129.55, 128.89, 128.85, 128.58, 128.45, 127.82, 127.78, 127.72, 116.32, 116.19, 116.11, 115.97. ^{19}F NMR (376 MHz, $CDCl_3$): δ (ppm) = -106.08, -132.29. ^{31}P NMR (162 MHz, $CDCl_3$): δ (ppm) = 28.23. HR Q-TOF MS: m/z calcd, 954.1128; found, 954.1125 [M].

Characterization of designed interlayer materials: ^{1}H, ^{13}C, ^{19}F, and ^{31}P nuclear magnetic resonance analyses were carried out on JEOL JNM-ECZ-400 NMR spectrometer. The mass spectra were acquired using Bruker Maxis-HD Ultra-High-Resolution Q-TOF MS system. Thermogravimetric analysis (TGA) was performed using a TGA Q500 V20.13 Build 39 at a heating rate of 10 °C min^{-1} under N_2 condition. UV-visible spectra were recorded on Perkin Elmer Lamda 365 UV-visible spectrometer. CV measurement was conducted with VersaSTAT 3 Potentiometry (Princeton Applied Research) with 0.1 M tetrabutylammonium hexafluorophosphate (TBAP) in acetonitrile in the presence of ferrocene/ferrocenium as an external standard. Glassy carbon electrodes, coated with small molecules, Ag wire, and Pt wire were used as a working electrode, reference electrode, and counter electrode, respectively.

Materials for Sn halide perovskite FETs: Phenethylammonium iodide (PEAI, > 95%) was purchased from GreatCell Solar Materials. Tin (II) iodide (SnI_2, 10-mesh beads, ultra-dry, 99.999%) was purchased from Alfa Aesar. Metallic tin (Sn powder, 99.5%), *N,N*-dimethylformamide (DMF, 99.8%, anhydrous), 1-methyl-2-pyrrolidinone (NMP, 99.5%, anhydrous), and chlorobenzene (CB, 99.8%,

anhydrous) were purchased from Sigma Aldrich. All the commercially available materials were used as received without further purification.

Perovskite precursor solution preparation: The PEA_2SnI_4 perovskite precursor solution (0.1 M) used in this work was prepared by dissolving PEAI and SnI_2 at a stoichiometric molar ratio of 2:1 into organic mixed solvent of DMF/NMP (3:1 vol ratio). Additionally, metallic Sn powder (5 mg ml^{-1}) was added to the perovskite precursor solution to prevent oxidation issue.[5,6] The precursor solution was stirred and heated at 60 °C for 4 h, and then cooled down to room temperature for usage. All the processes for the solution preparation were carried out in a N_2-filled glovebox with oxygen and moisture levels lower than 1.0 ppm.

FET fabrication: Heavily doped silicon wafer substrate (0.001 Ω cm^{-1}) with thermally grown SiO_2 dielectric layer (300 nm thickness, and capacitance of approximately 11.3 nF cm^{-2}) was employed for bottom-gate top-contact FETs. The substrate was first cleaned in sonication bath with deionized water, acetone, and isopropanol for 15 min in sequence, and then dried with N_2 gas flow. After drying, the substrate was subjected to UV-ozone treatment for 10 min and then transferred into a N_2-filled glovebox for film deposition. The as-prepared fresh PEA_2SnI_4 precursor solution was filtered through a 0.2 μm PTFE filter and spin-coated onto the substrate at 4000 rpm for 30 s, followed by annealing at 100 °C for 10 min. To introduce a passivation layer, the solution of synthesized molecule (dissolved into 1 mL CB with 0.25 mg) was deposited using the spin coating method on the perovskite layer (7500 rpm, 40 s) with the thickness of around 5 nm (measured by ellipsometer), and annealed (100 °C for 5 min). This was followed by high vacuum thermal evaporation (2.0×10^{-6} Torr at a deposition rate of 0.2 Å s^{-1}) of the Au source and drain electrodes with shadow metal mask (channel width = 1,500 μm, and length = 100 μm). Finally, the device active area went through a glass encapsulation process to prevent oxidation issue.

Perovskite film and device characterizations: Scanning electron microscopy (FE-SEM, S-4800, Hitachi) was used to characterize the morphology of the perovskite thin films. X-ray diffraction (XRD) spectra of the perovskite films were recorded by an X-ray diffractometer (D8 Advance, Bruker) with a Cu Kα irradiation (λ = 1.5406 Å). The thin film XRD patterns were scanned in the 2θ between 2° and 40°, with a step of 0.02°. The distance of interlayer was calculated by Bragg's law: $2d\sin\theta = n\lambda$, where λ is the X-ray wavelength, 1.5418 Å, θ is the Bragg angle of the diffraction peak, and $n = 2$, respectively. A UV-visible spectrometer (UV-2600, SCINCO) was utilized for collecting the optical absorbance spectra of the thin films in the range of 300-800 nm at room temperature. Photoluminescence (PL) measurements were conducted at room temperature using PL spectrophotometer (FlouTime 300, PicoQuant Co.), using an excitation light source with a wavelength of 410 nm, and the spectra were measured in the 500-850 nm. ^{31}P nuclear magnetic resonance analysis was carried out on VNMRS 600MHz NMR spectrometer, VARIAN. FT-IR spectra were recorded on a Nicolet6700 FTIR spectrometer (Thermo Scientific) with an attenuated total reflectance (ATR) mode. Kelvin probe force microscope (KPFM) measurements were performed on commercial AFM (NX20, Park systems) for scanning the in-situ surface potential distributions with Au-coated Cr tip (PPP-NSC36). All samples were scanned in an area of 5 μm × 5 μm, with a scan rate 0.3 Hz. The values of work function (WF) and valence band maximum (VBM) of all prepared perovskite thin films

were determined using ultraviolet photoemission spectroscopy (UPS, XPS-Theta Probe, Thermo Fisher Scientific Co.) with He I (21.2 eV) as the excitation UV source. The UPS measurement data of Au top electrodes with and without 2F-TPOF2 are represented in Figure S32 (Supporting Information) for reference. The contact angle measurement system (CA-500A, IRASOL) was utilized to determine the water contact angles of perovskite thin films. X-ray photoelectron spectroscopy (XPS) spectra were recorded with Al Kα X-ray source (K-alpha plus, Thermo Fisher Scient) (12 keV/6 mA, 400 μm). All electrical measurements, except the storage stability test, were performed using a probe station placed in the dark shield box under ambient conditions and room temperature (a relative humidity 35-45 %). The probe station was connected to a semiconductor parameter analyzer (Keithley 2636B).

Supplementary Note 1. Methods to extract mobility values of PEA_2SnI_4 FETs.

Threshold voltage (V_{th}) and claimed carrier mobility (μ_{claimed}) values are determined from the forward transfer characteristic curves in the saturation regime (Figure S30, Supporting Information) using the following equations[7]:

$$I_{\mathrm{DS,sat}} = \frac{\mu_{\mathrm{sat}} W C_{\mathrm{i}}}{2L}(V_{\mathrm{GS}} - V_{\mathrm{th}})^2,\ |V_{\mathrm{GS}} - V_{\mathrm{th}}| < |V_{\mathrm{DS}}| \quad (1)$$

$$\mu_{\mathrm{sat}} = \frac{2L}{WC_{\mathrm{i}}}\left(\frac{\partial\sqrt{|I_{\mathrm{DS}}|}}{\partial V_{\mathrm{GS}}}\right)^2 \quad (2)$$

, where L, W, and C_i represent the channel length, channel width, and specific dielectric capacitance (per unit area), respectively. I_{DS} and V_{GS} denote the source-drain current and gate-source voltage, respectively.

Given the influence of various intrinsic and extrinsic factors, such as gate dielectric inhomogeneity, ion migration, charge trapping, and scattering, careful consideration is essential when extracting accurate mobility values. For a more reliable assessment, we calculated the measurement reliability factor (r_{sat}) for the FET devices from the forward transfer characteristics in the saturation regime (Figure S30, Supporting Information), and estimated the effective mobility ($\mu_{\mathrm{effective}}$) to ensure the overall validity of the mobility values reported in this manuscript[8]:

$$r_{\mathrm{sat}} = \left(\frac{\sqrt{|I_{\mathrm{DS}}|^{\mathrm{max}}} - \sqrt{|I_{\mathrm{DS}}^0|}}{|V_{\mathrm{GS}}|^{\mathrm{max}}}\right)^2 \Bigg/ \left(\frac{\partial\sqrt{|I_{\mathrm{DS}}|}}{\partial V_{\mathrm{GS}}}\right)^2_{\mathrm{claimed}} \quad (3)$$

$$\mu_{\mathrm{effective}} = r_{\mathrm{sat}} \times \mu_{\mathrm{claimed}} \quad (4)$$

, where $|I_{\mathrm{DS}}|^{\mathrm{max}}$ and $|I_{\mathrm{DS}}^0|$ denote the experimental maximum source-drain current at the maximum gate voltage $|V_{\mathrm{GS}}|^{\mathrm{max}}$ (-40 V) and the source-drain current at V_{GS} = 0 V, respectively. All μ_{claimed} and r_{sat} values for $\mu_{\mathrm{effective}}$ calculations are provided in Table S4 (Supporting Information), and the associated double-linear transfer curves are presented in Figure S30 (Supporting Information).

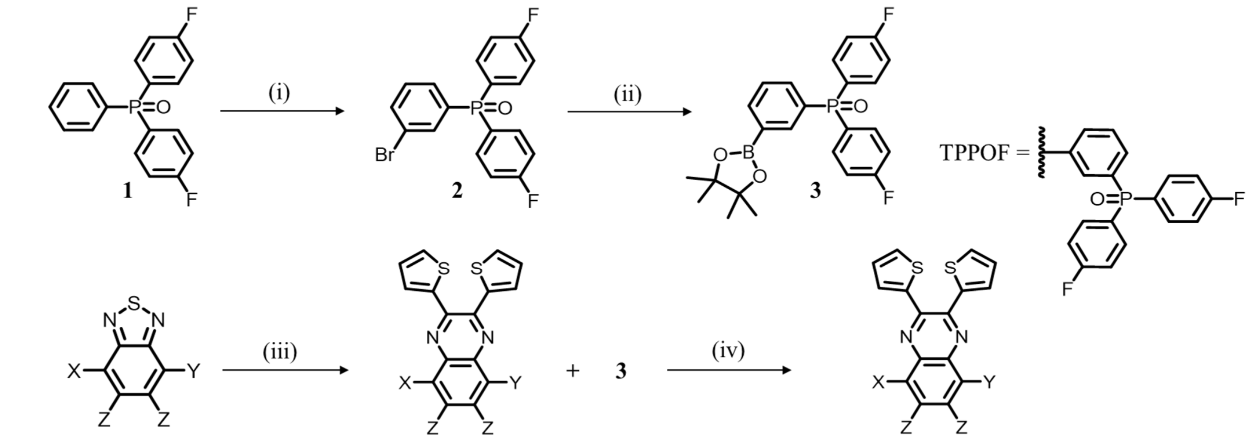

4 : X = H, Y = Br, Z = H
5 : X = Br, Y = Br, Z = H
6 : X = Br, Y = Br, Z = F

7 : X = H, Y = Br, Z = H
8 : X = Br, Y = Br, Z = H
9 : X = Br, Y = Br, Z = F

TPOF1 : X = H, Y = TPPOF, Z = H
TPOF2 : X = TPPOF, Y = TPPOF, Z = H
2F-TPOF2 : X = TPPOF, Y = TPPOF, Z = F

Figure S1. Synthetic procedures for QPSMs: (i) *N*-bromosuccinimde/H_2SO_4 under N_2 at room temperature for 4 days; (ii) Bis(pinacolato)diboron/Pd(dppf)Cl_2/CH_3COOK/toluene under N_2 at 100 °C for 24 h; (iii) Zinc/acetic acid at 85 °C for 1 h and then 2,2'-thenil/acetic acid at 110 °C for overnight; (iv) Pd(PPh_3)$_4$/2M K_2CO_3/toluene at 90 °C for 48 h.

a

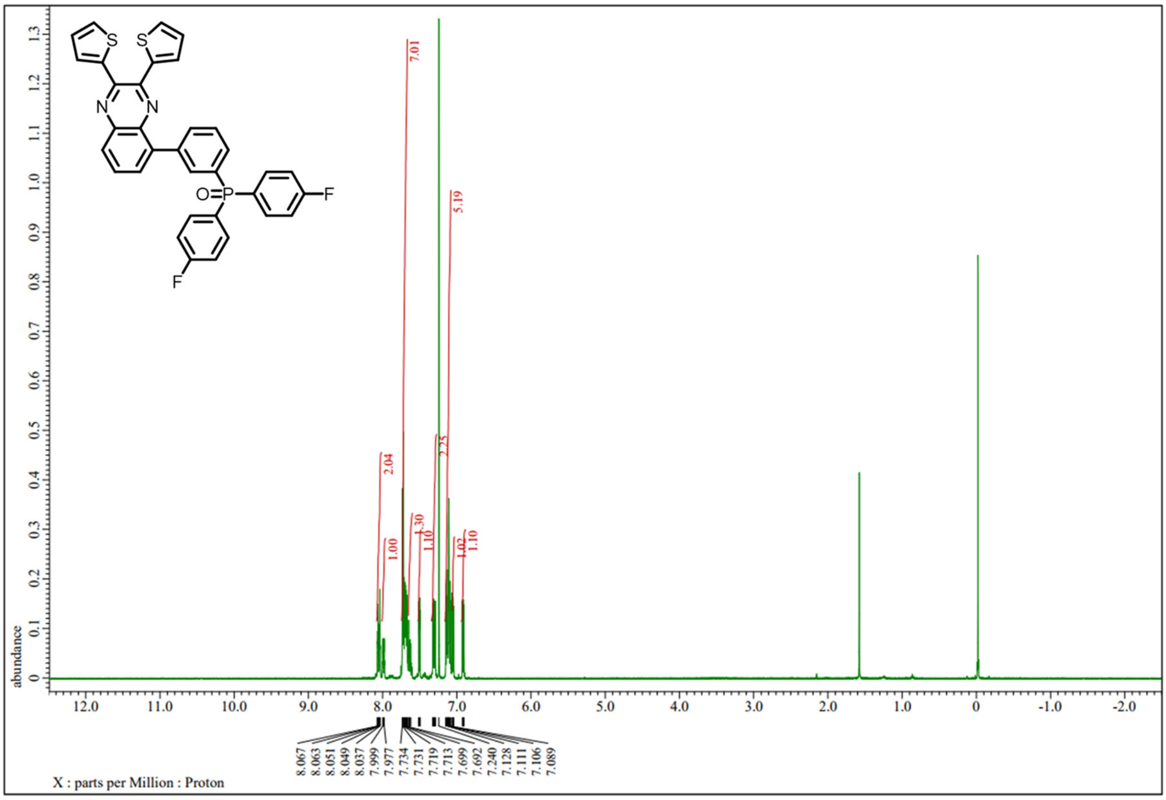

b

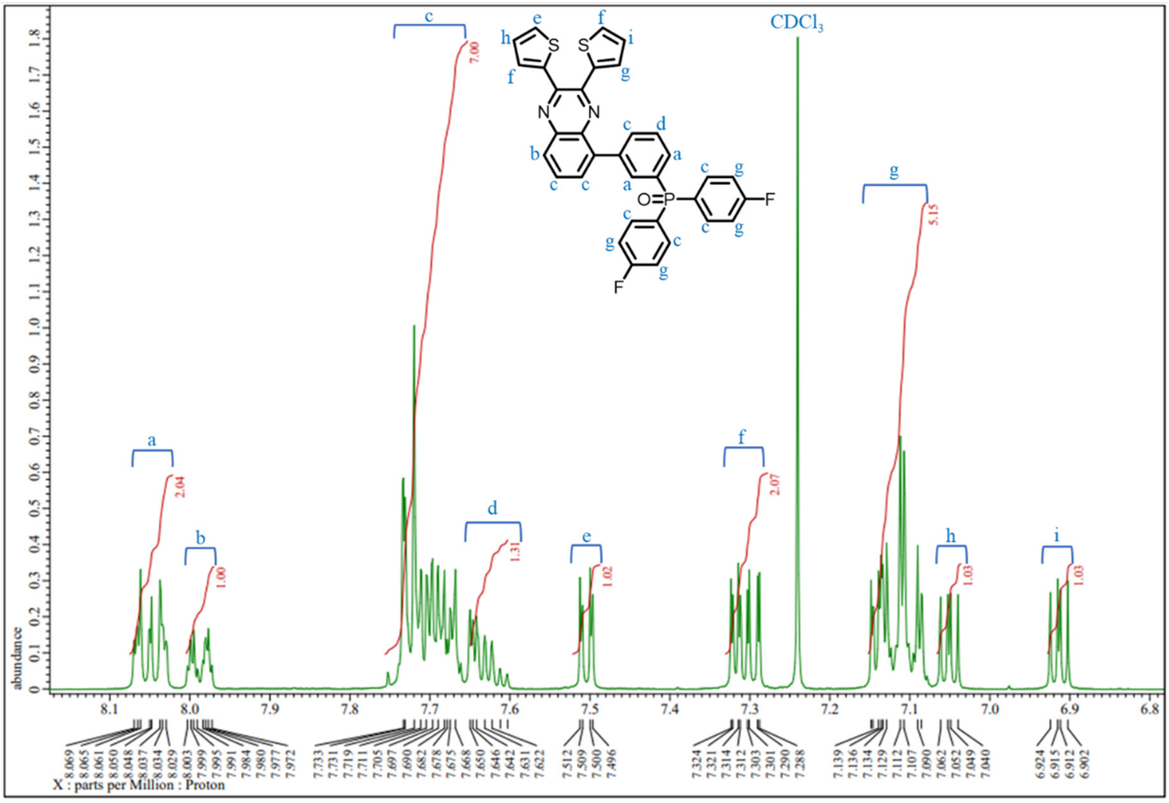

Figure S2. a ^{1}H NMR spectrum of TPOF1. **b** Enlarge spectrum with interpretation.

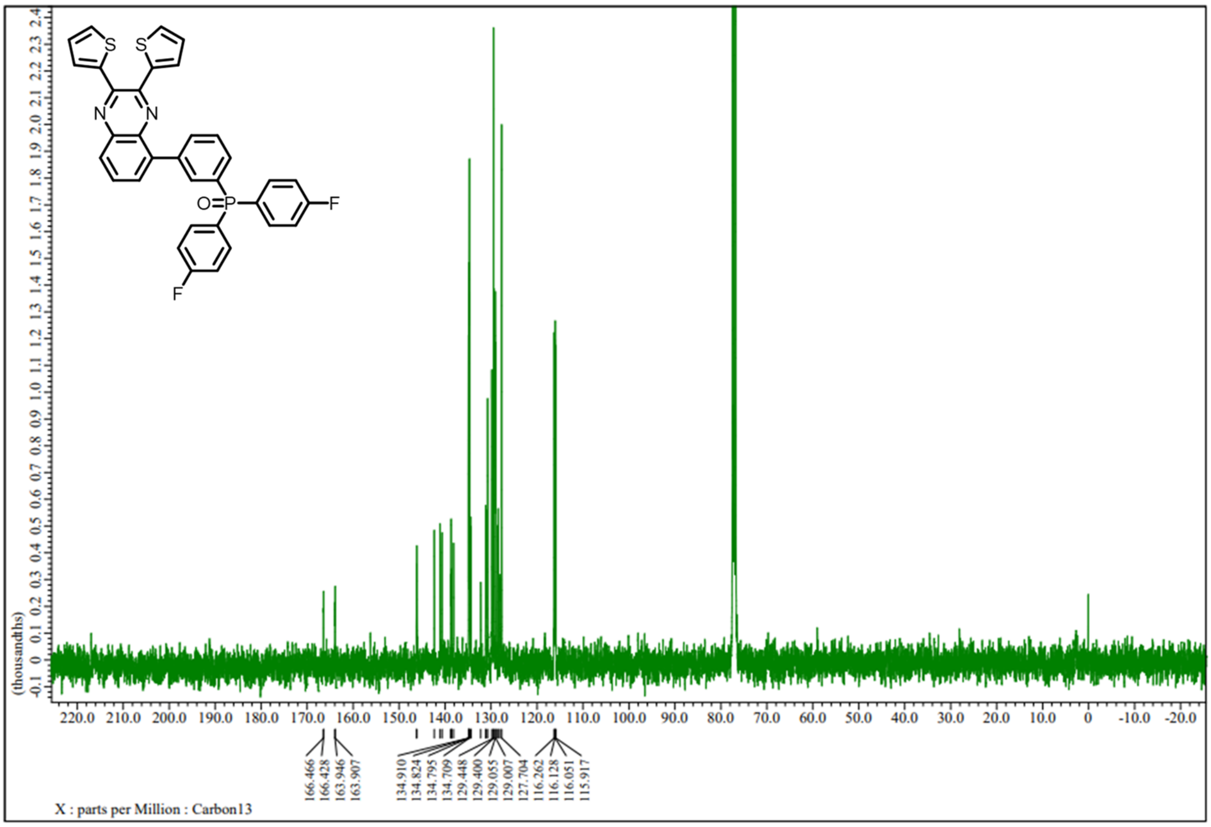

Figure S3. ^{13}C NMR spectrum of TPOF1.

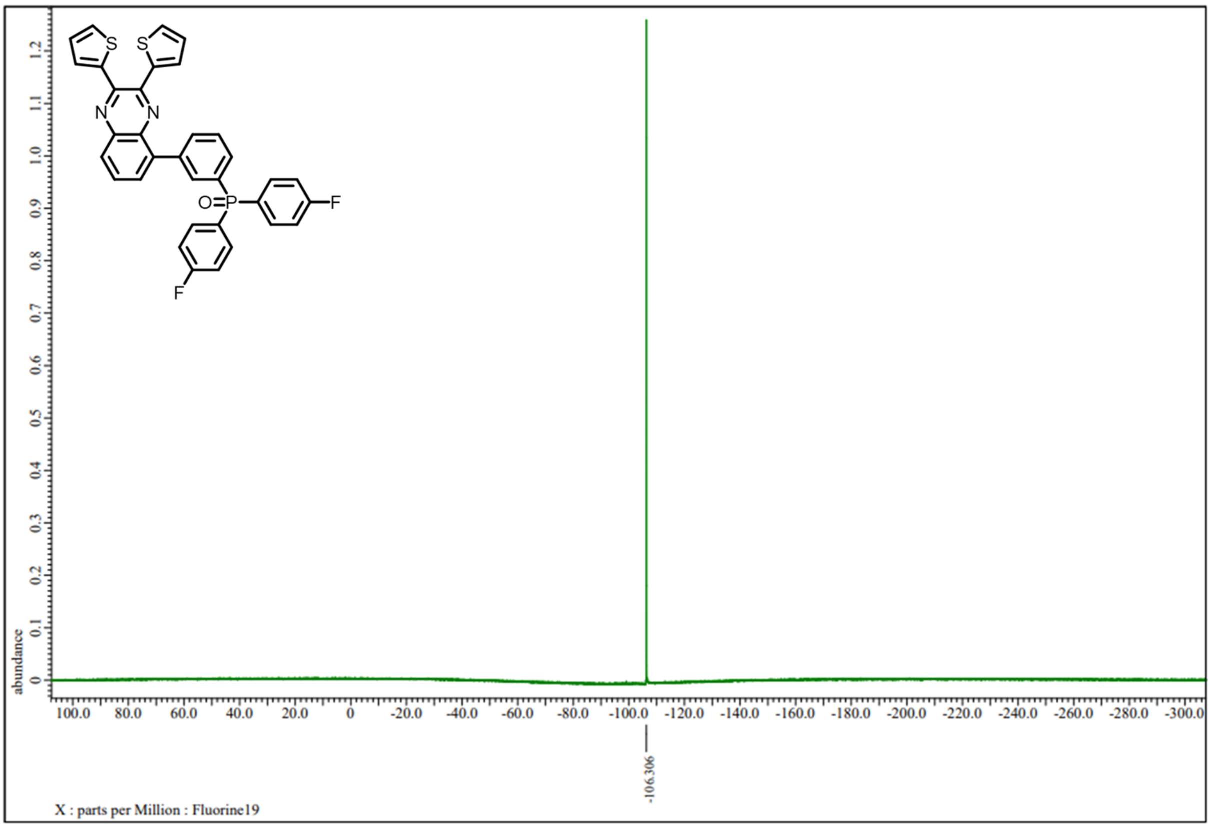

Figure S4. ^{19}F NMR spectrum of TPOF1.

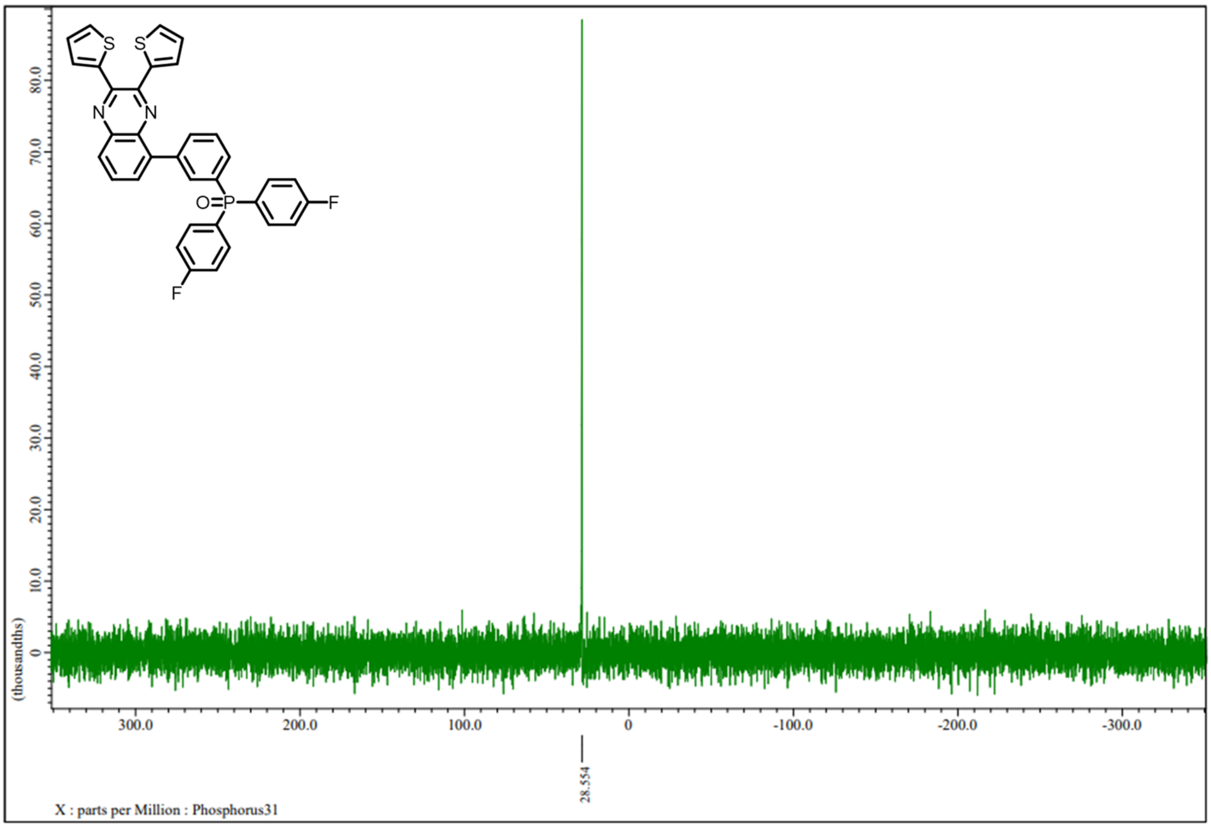

Figure S5. ^{31}P NMR spectrum of TPOF1.

a

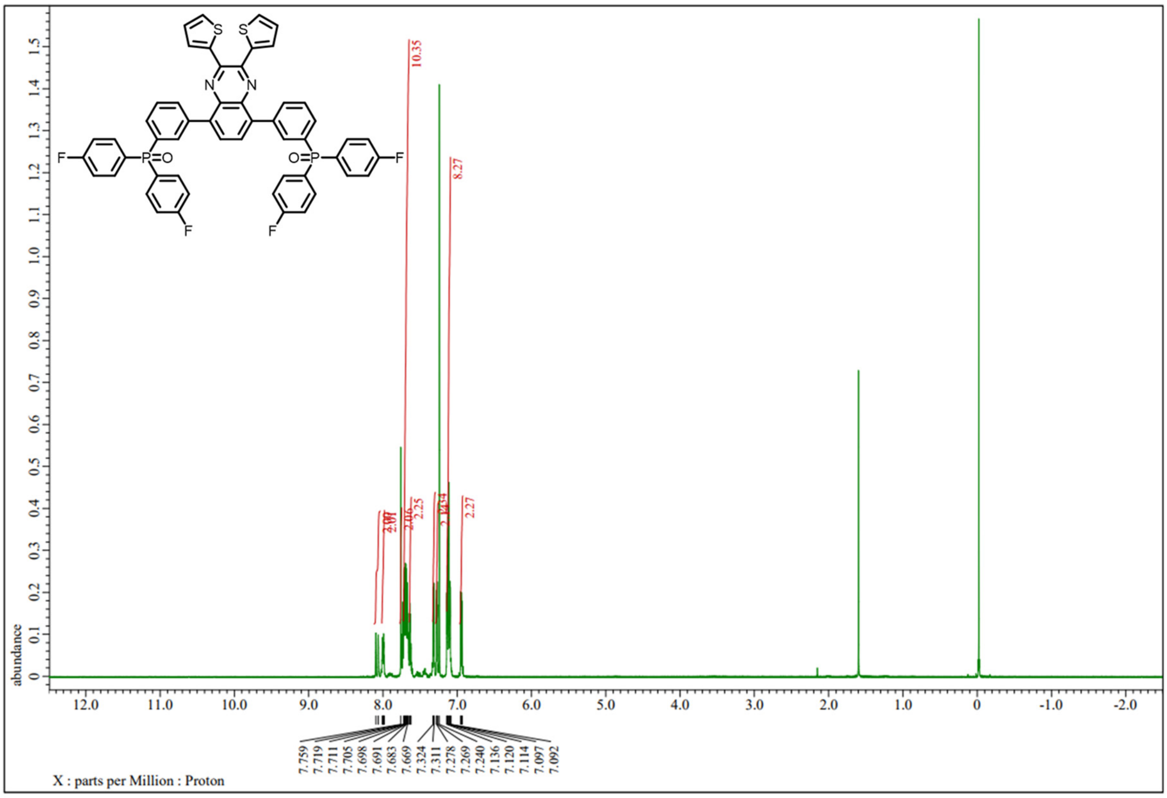

b

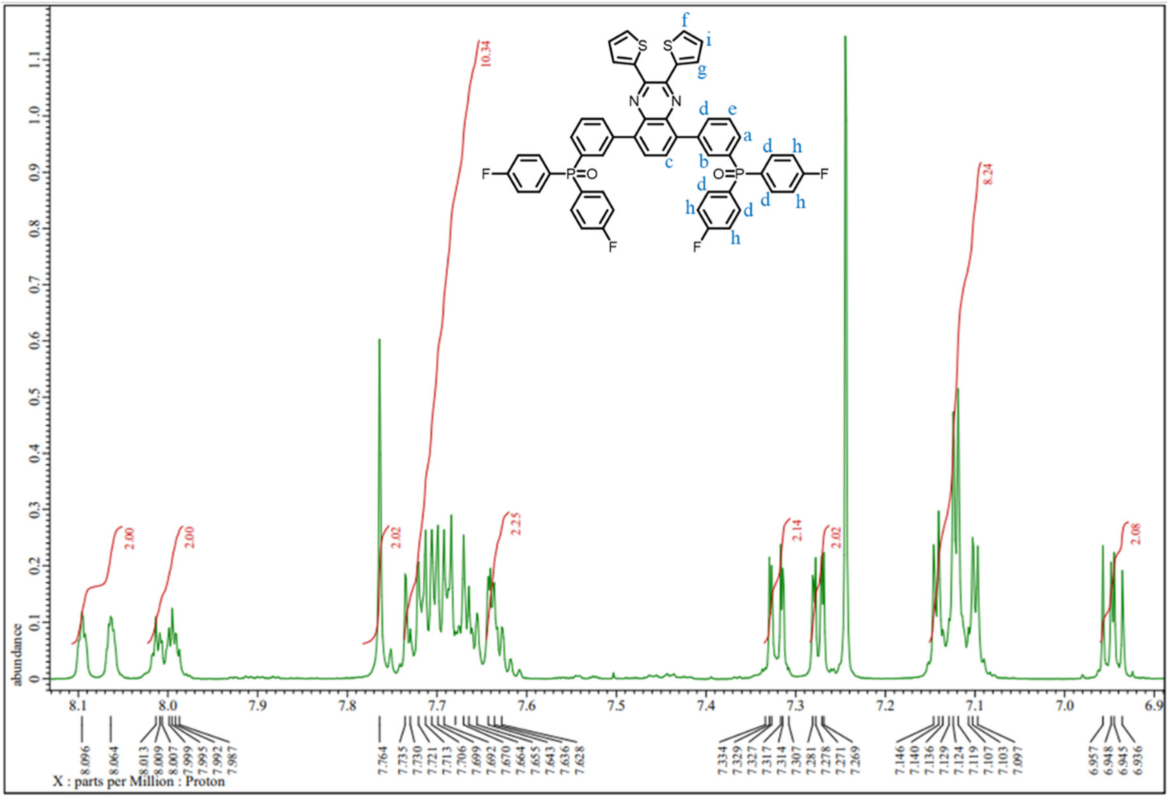

Figure S6. a ^{1}H NMR spectrum of TPOF2. **b** Enlarge spectrum with interpretation.

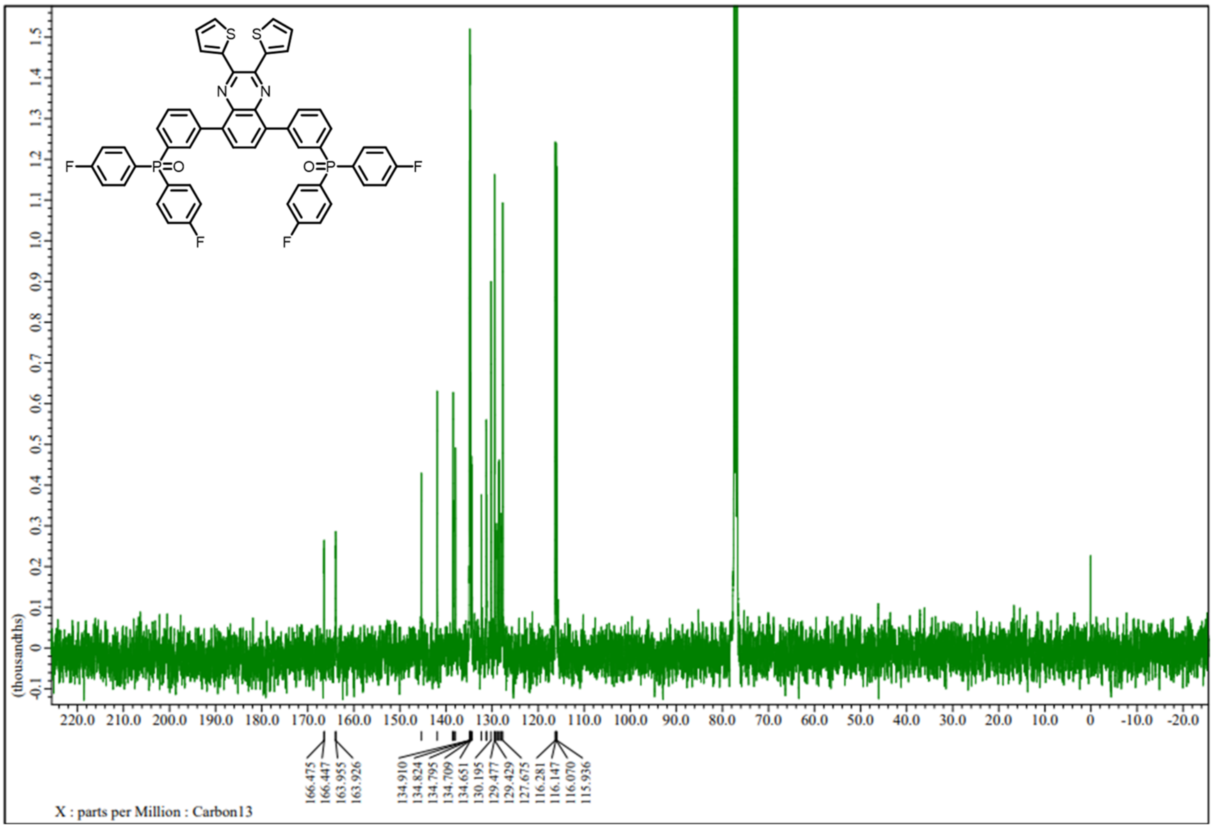

Figure S7. ^{13}C NMR spectrum of TPOF2.

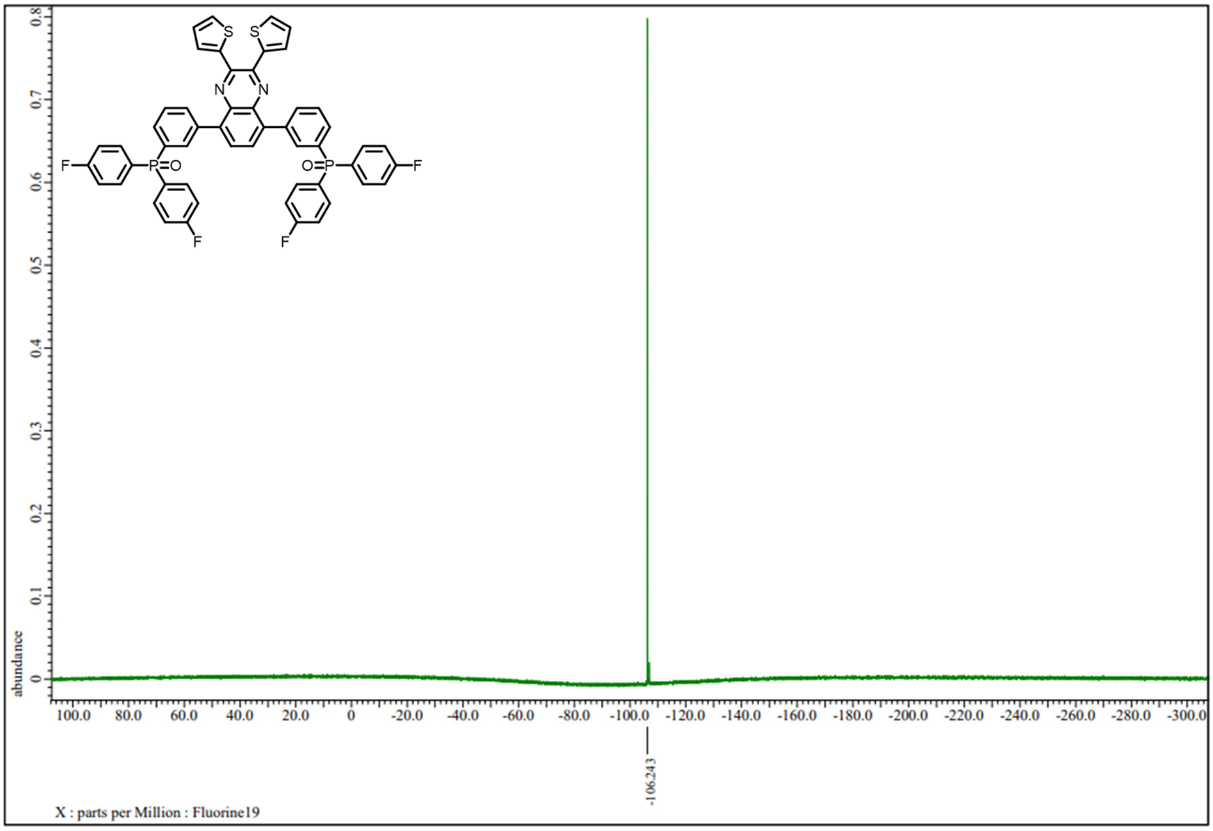

Figure S8. ^{19}F NMR spectrum of TPOF2.

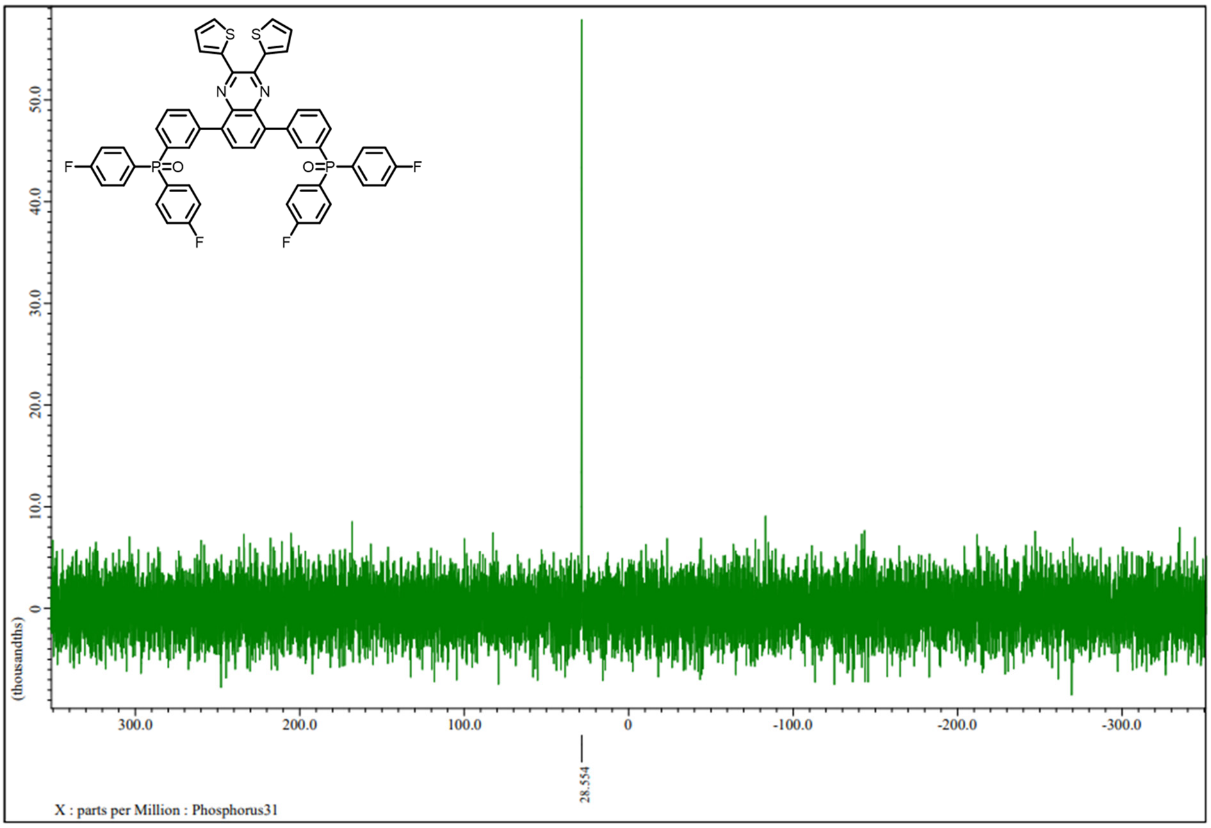

Figure S9. ^{31}P NMR spectrum of TPOF2.

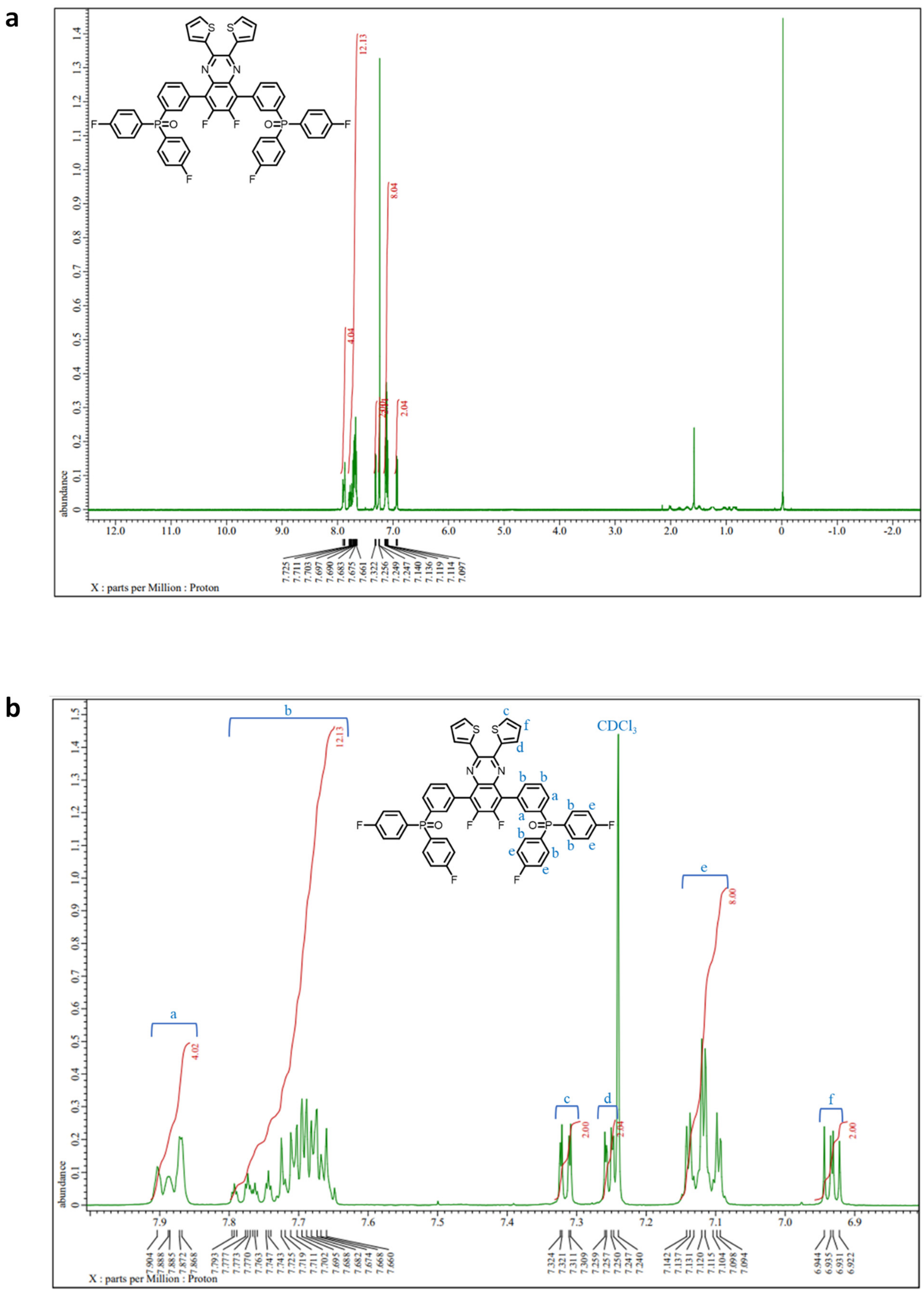

Figure S10. a ^{1}H NMR spectrum of 2F-TPOF2. **b** Enlarge spectrum with interpretation.

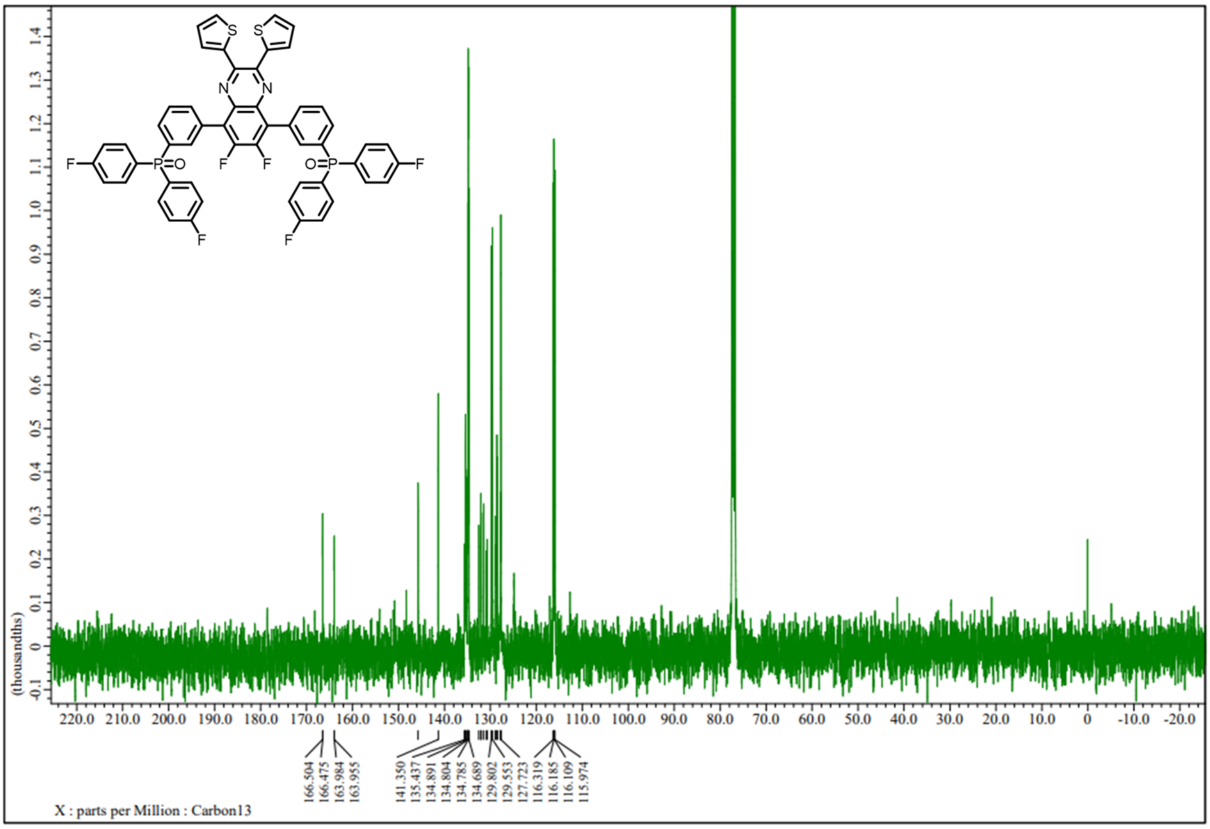

Figure S11. ^{13}C NMR spectrum of 2F-TPOF2.

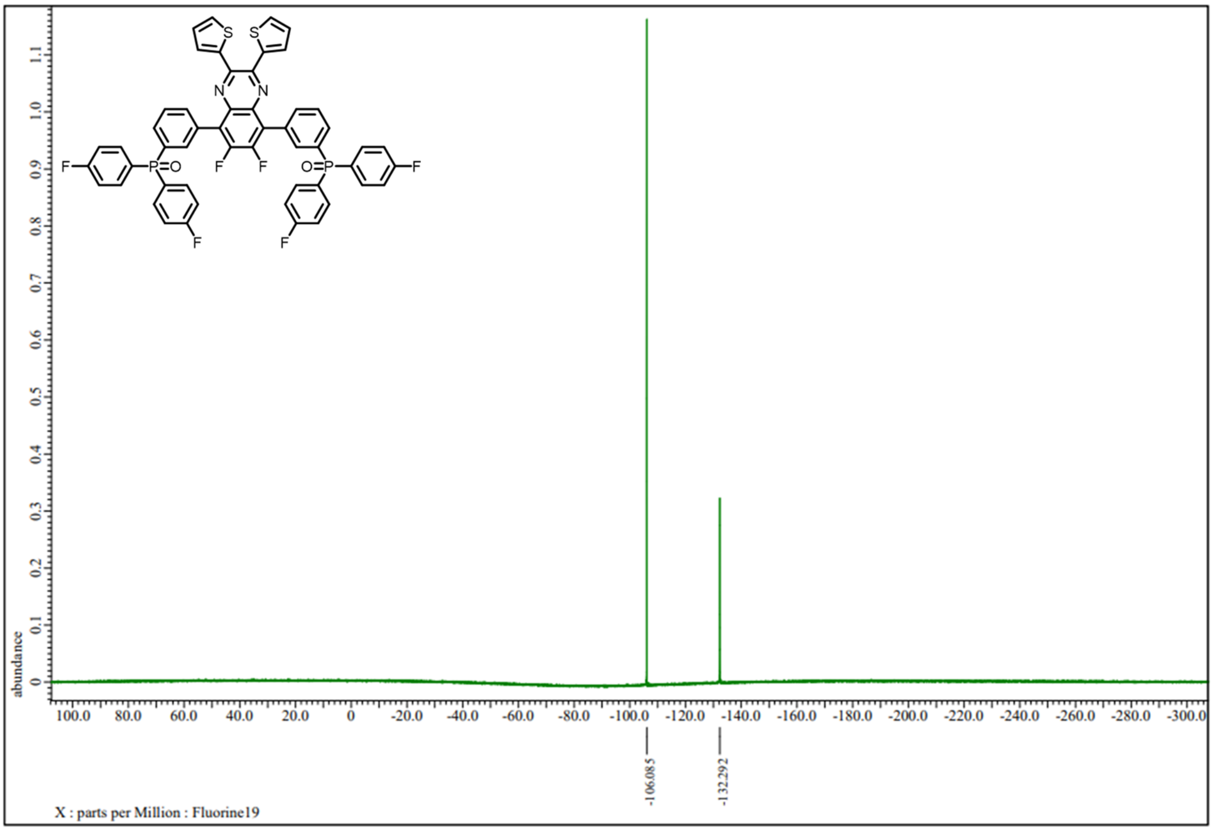

Figure S12. ^{19}F NMR spectrum of 2F-TPOF2.

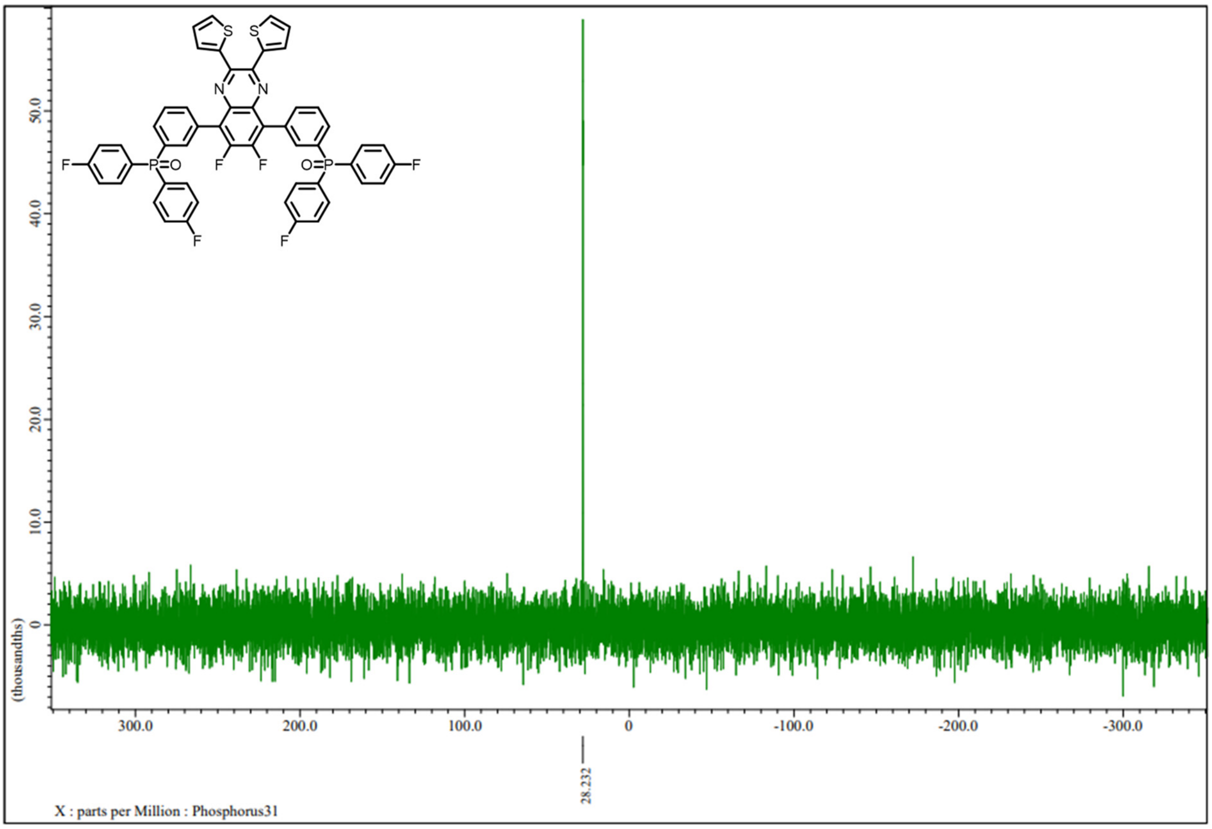

Figure S13. ^{31}P NMR spectrum of 2F-TPOF2.

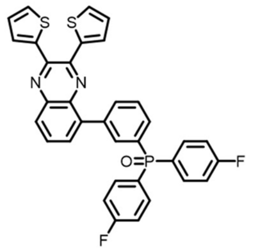

Exact Mass: 606.0801
m/z: 606.0801 (100.0%), 607.0835 (36.8%), 608.0759 (9.0%), 608.0868 (6.6%), 609.0792 (3.3%), 607.0795 (1.6%)

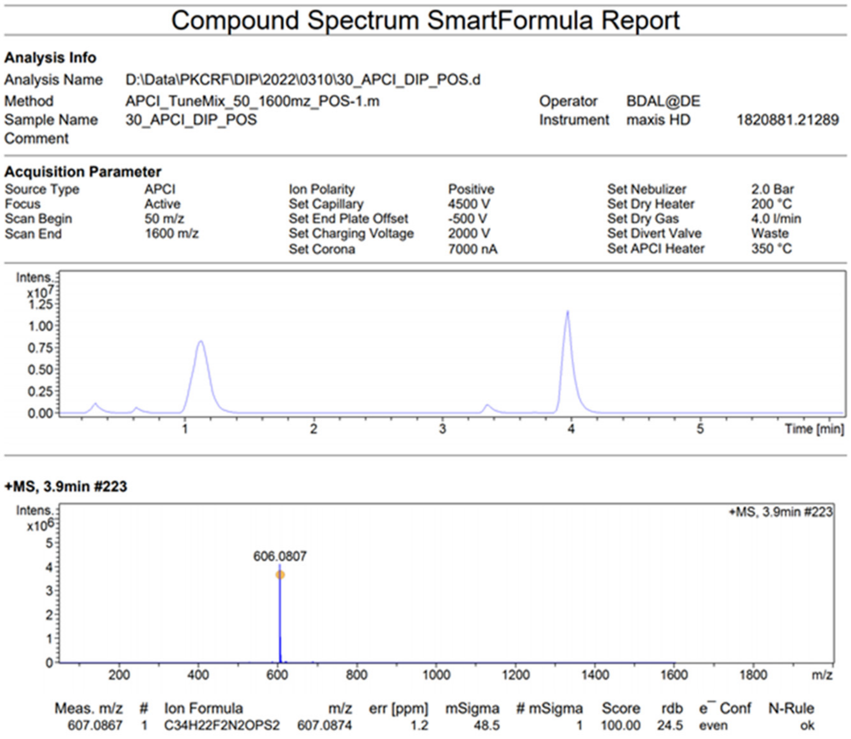

Figure S14. Mass spectroscopic analysis spectrum of TPOF1.

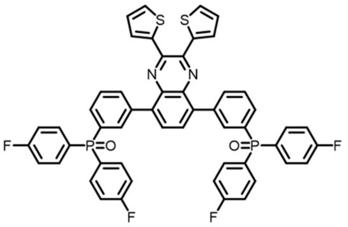

Exact Mass: 918.1317
m/z: 918.1317 (100.0%), 919.1350 (56.2%), 920.1384 (15.5%), 920.1275 (9.0%), 921.1308 (5.1%), 921.1417 (2.8%), 919.1310 (1.6%), 922.1342 (1.4%)

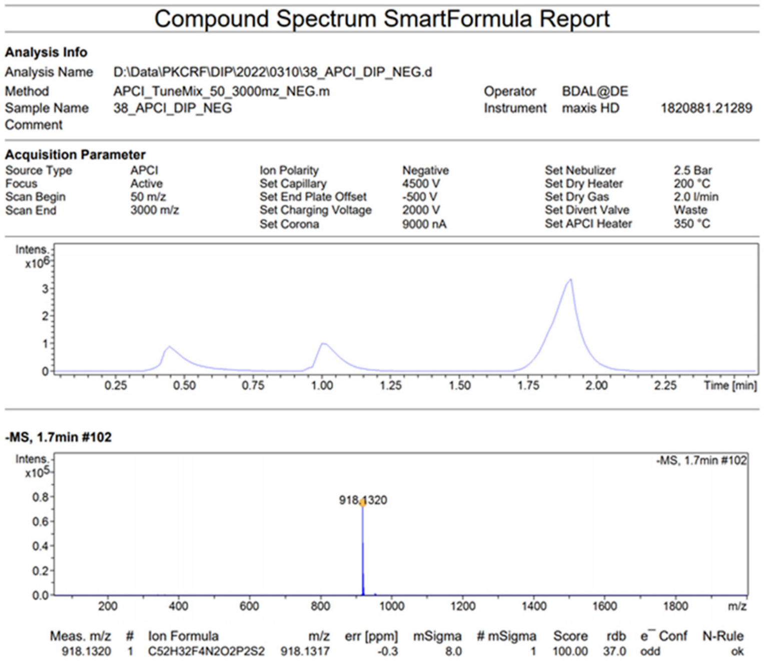

Figure S15. Mass spectroscopic analysis spectrum of TPOF2.

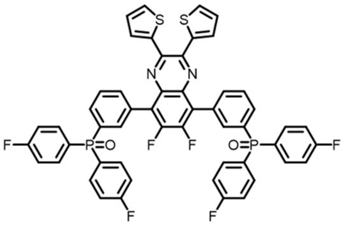

Exact Mass: 954.1128
m/z: 954.1128 (100.0%), 955.1162 (56.2%), 956.1195 (15.5%), 956.1086 (9.0%), 957.1120 (5.1%), 957.1229 (2.0%), 955.1122 (1.6%), 958.1153 (1.4%)

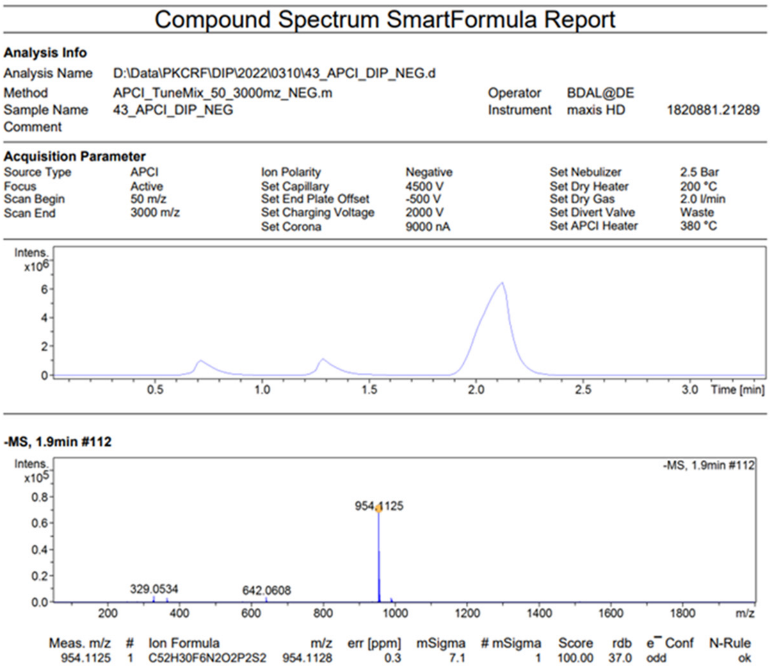

Figure S16. Mass spectroscopic analysis spectrum of 2F-TPOF2.

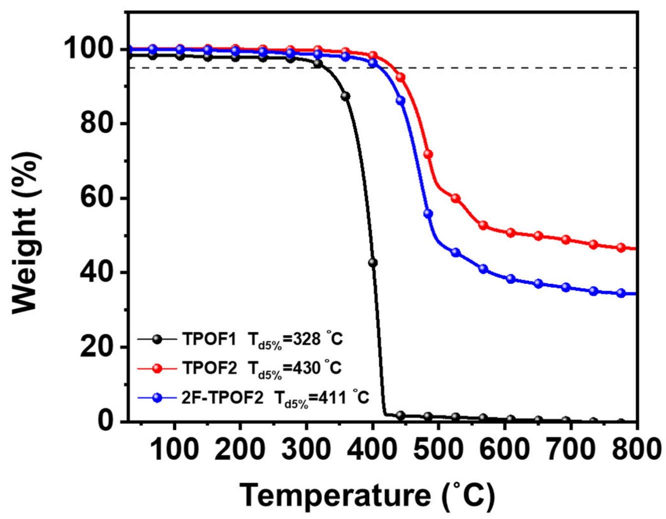

Figure S17. Thermogravimetric analysis results of the designed molecules.

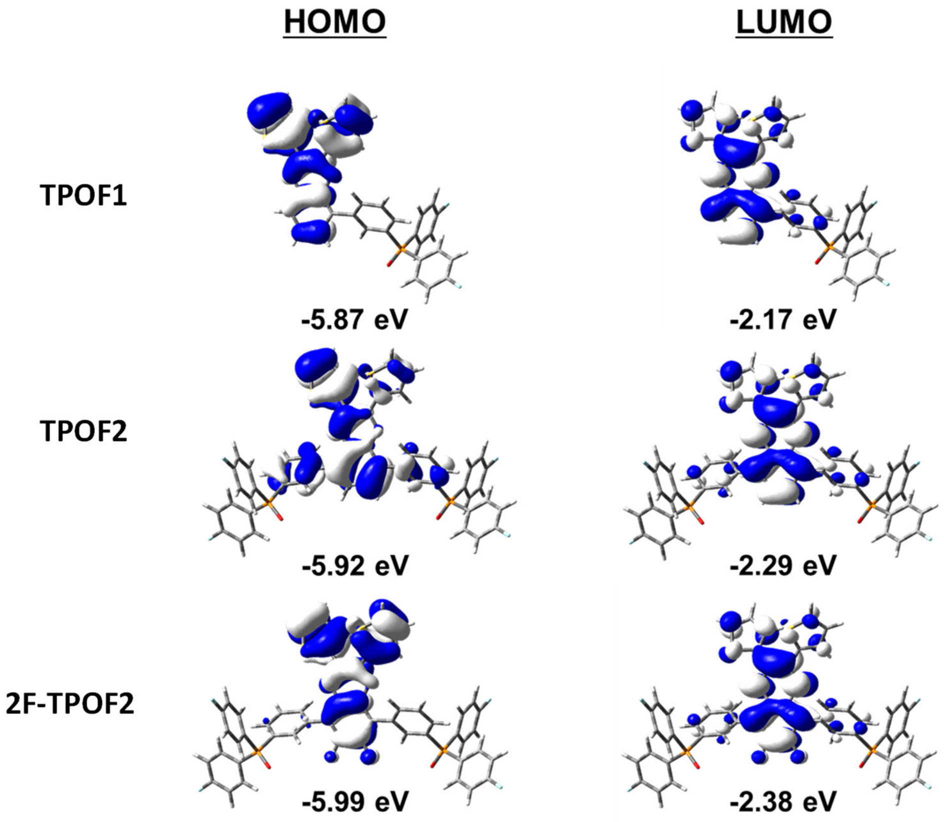

Figure S18. Orbital diagrams (electron density plots) of the designed molecules, calculated by DFT. Theoretically calculated energy band levels are added.

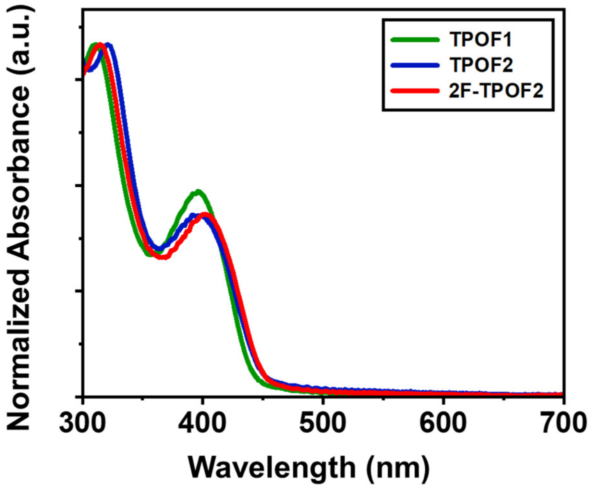

Figure S19. UV-visible spectra of TPOF1, TPOF2, and 2F-TPOF2 thin films casted on quartz substrates.

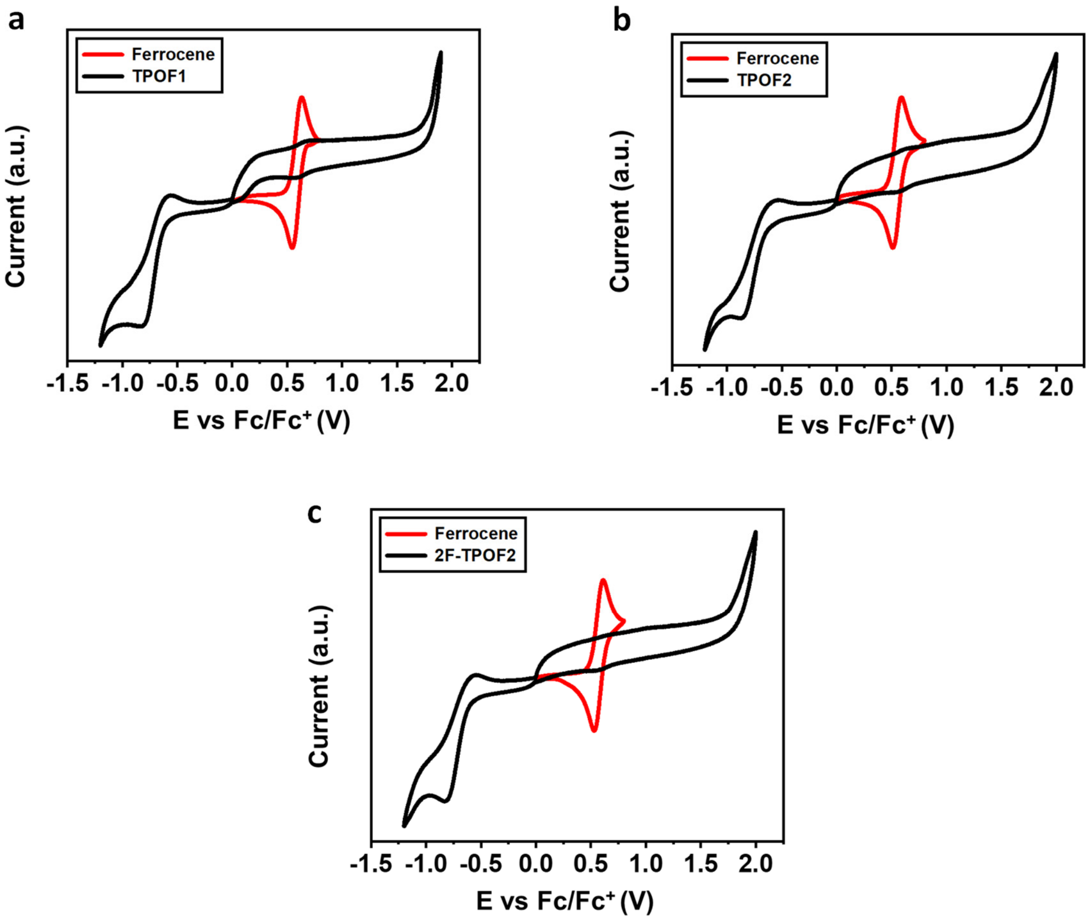

Figure S20. Cyclic voltammetry plots of the designed molecules (black solid line), and ferrocene (red solid line): **a** TPOF1, **b** TPOF2, and **c** 2F-TPOF2.

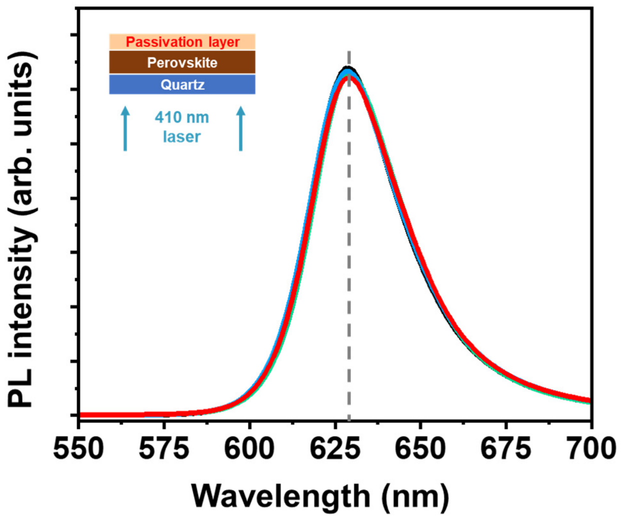

Figure S21. Steady-state PL emission spectra of PEA_2SnI_4 films casted on quartz substrates with and without molecular interlayer: 410 nm wavelength laser excitation from bottom side (quartz substrate). Black, green, blue, and red colors represent pristine PEA_2SnI_4, PEA_2SnI_4 with TPOF1, PEA_2SnI_4 with TPOF2, and PEA_2SnI_4 with 2F-TPOF2, respectively.

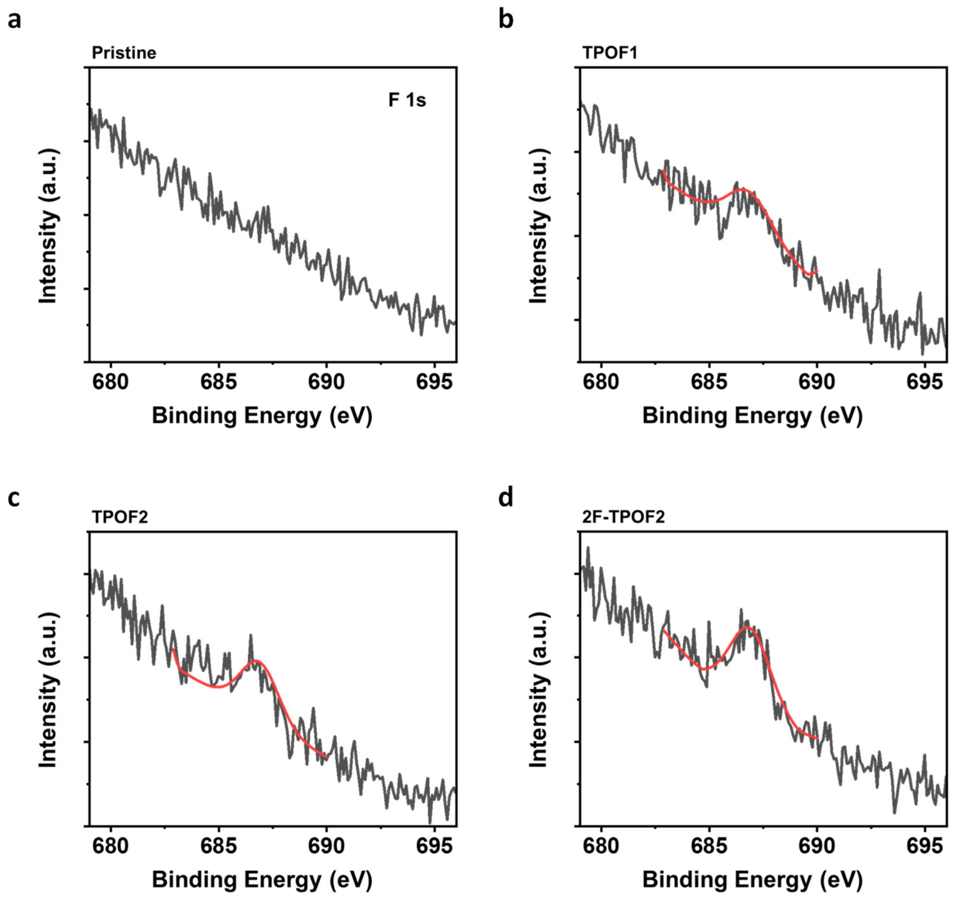

Figure S22. XPS F 1s peaks of PEA_2SnI_4 thin films with and without molecular interlayers: **a** pristine PEA_2SnI_4, **b** PEA_2SnI_4 with TPOF1, **c** PEA_2SnI_4 with TPOF2, and **d** PEA_2SnI_4 with 2F-TPOF2.

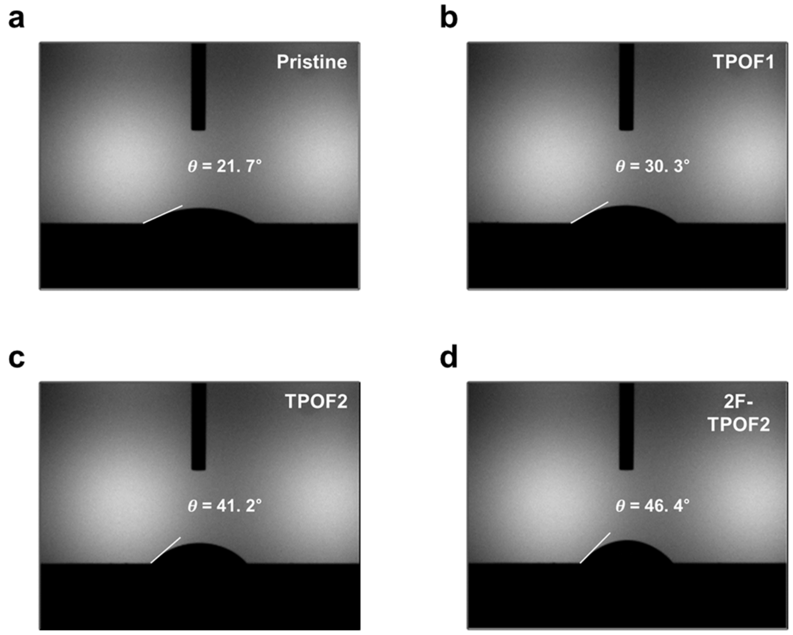

Figure S23. Water contact angle analyses of the PEA_2SnI_4 films with and without molecular interlayers: **a** pristine PEA_2SnI_4, **b** PEA_2SnI_4 with TPOF1, **c** PEA_2SnI_4 with TPOF2, and **d** PEA_2SnI_4 with 2F-TPOF2.

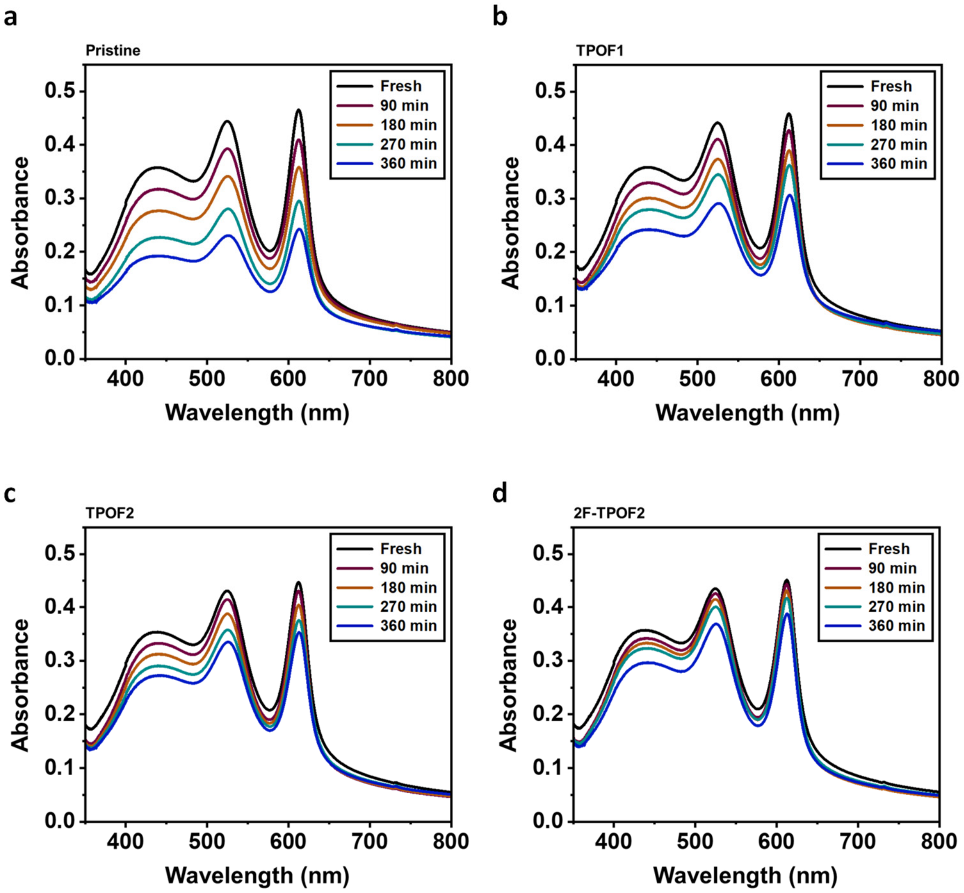

Figure S24. UV-visible absorbance spectra of PEA_2SnI_4 films casted on quartz substrates with and without molecular interlayers at various air exposure times from 0 min (fresh) up to 360 min: **a** pristine PEA_2SnI_4, **b** PEA_2SnI_4 with TPOF1, **c** PEA_2SnI_4 with TPOF2, and **d** PEA_2SnI_4 with 2F-TPOF2.

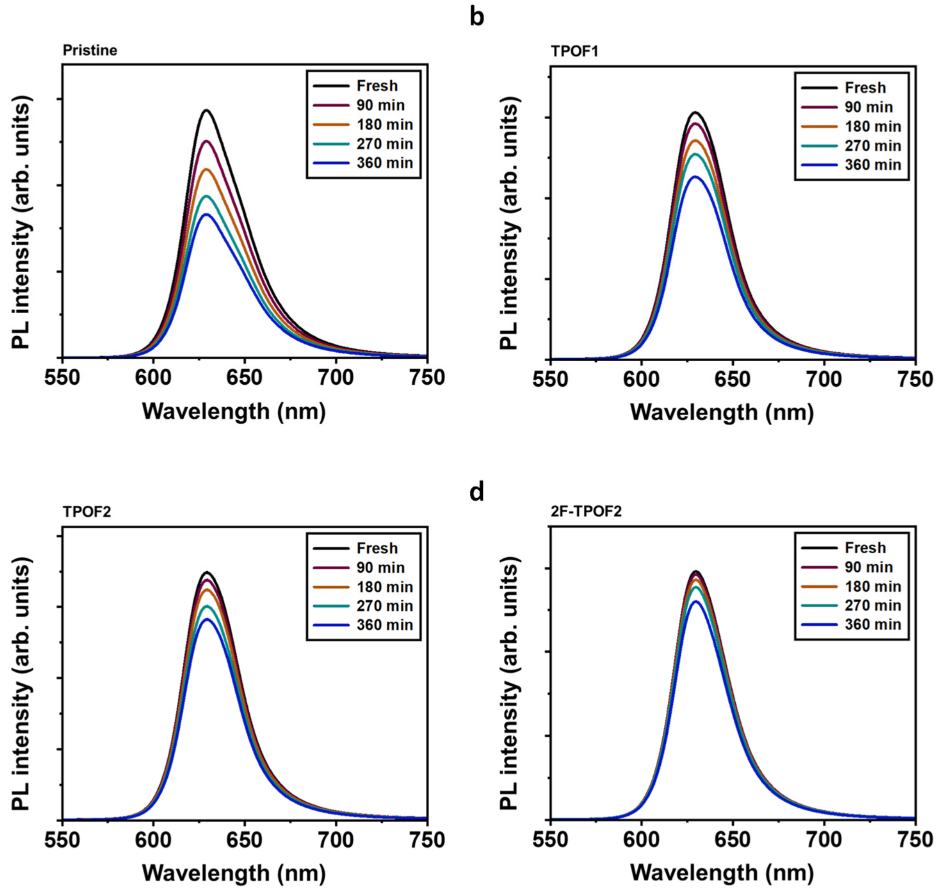

Figure S25. Steady-state PL spectra of PEA_2SnI_4 films casted on quartz substrates with and without molecular interlayers at various air exposure times from 0 min (fresh) up to 360 min. 410 nm wavelength laser excitation from top side (molecular interlayer): **a** pristine PEA_2SnI_4, **b** PEA_2SnI_4 with TPOF1, **c** PEA_2SnI_4 with TPOF2, and **d** PEA_2SnI_4 with 2F-TPOF2.

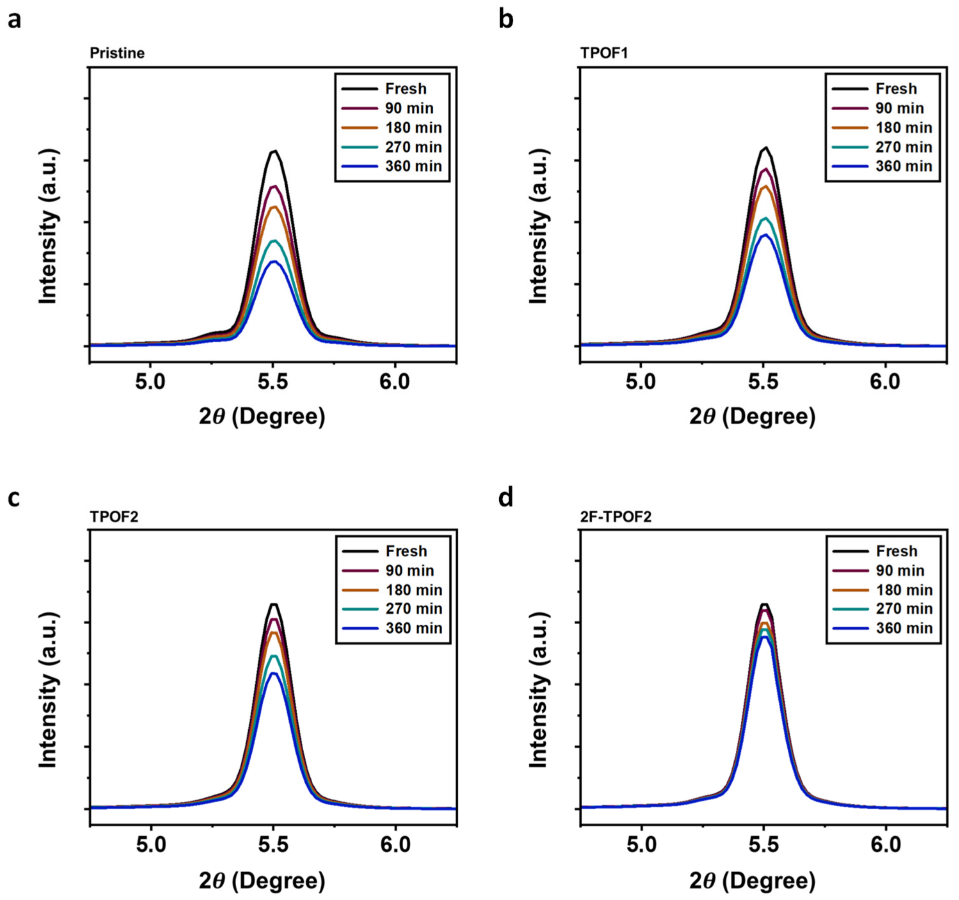

Figure S26. High-magnification XRD patterns of PEA_2SnI_4 films with and without molecular interlayers around intense peak of 5.4° under the various air exposure times from 0 min (fresh) to 360 min: **a** pristine PEA_2SnI_4, **b** PEA_2SnI_4 with TPOF1, **c** PEA_2SnI_4 with TPOF2, and **d** PEA_2SnI_4 with 2F-TPOF2.

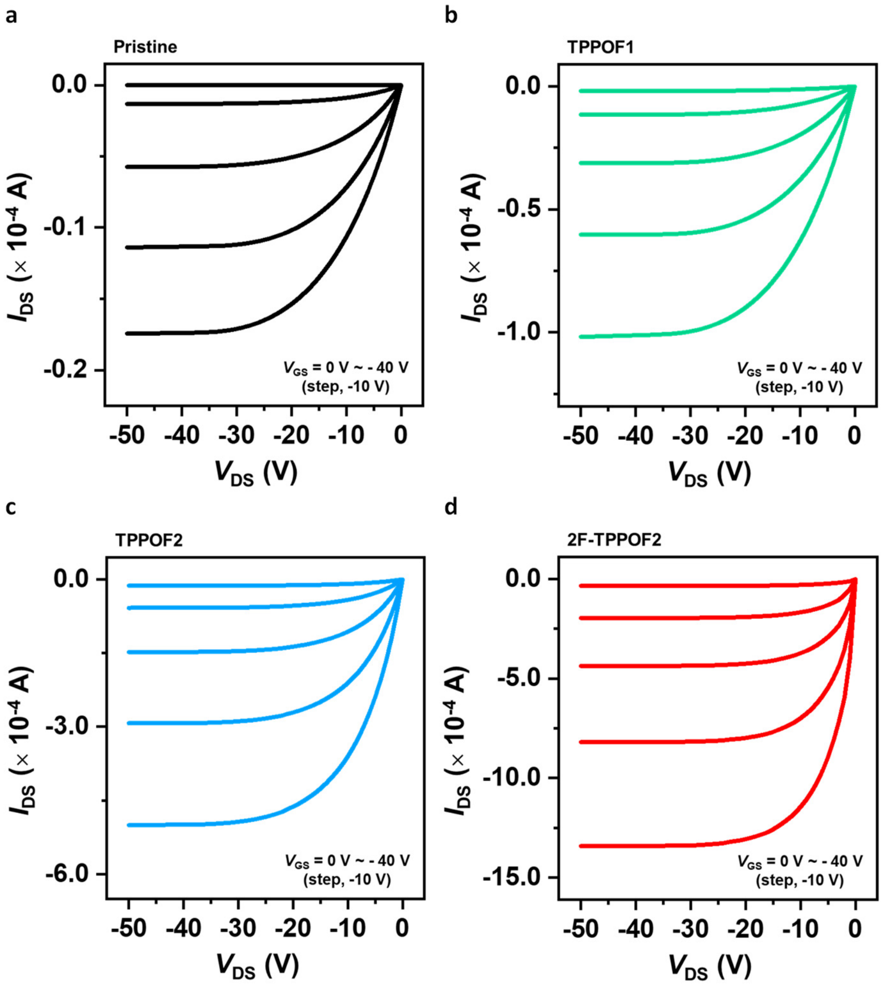

Figure S27. Output characteristics of PEA_2SnI_4 FETs with and without molecular interlayers at various V_{GS} conditions (V_{GS} = 0, -10, -20, -30, and -40 V): **a** pristine PEA_2SnI_4, **b** PEA_2SnI_4 with TPOF1, **c** PEA_2SnI_4 with TPOF2, and **d** PEA_2SnI_4 with 2F-TPOF2.

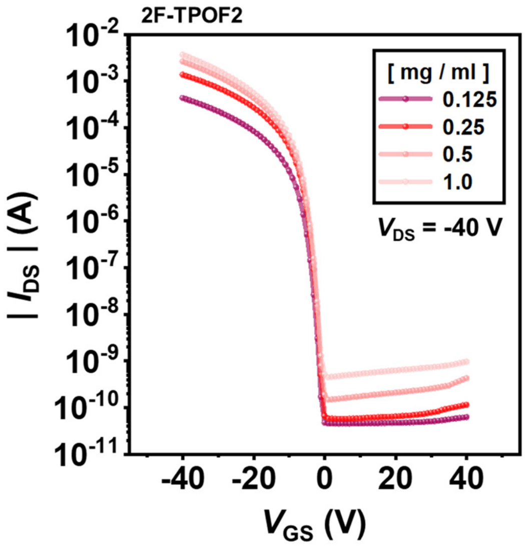

Figure S28. Transfer characteristics for forward scan of PEA_2SnI_4 FET devices as a function of 2F-TPOF2 molecular interlayer thickness.

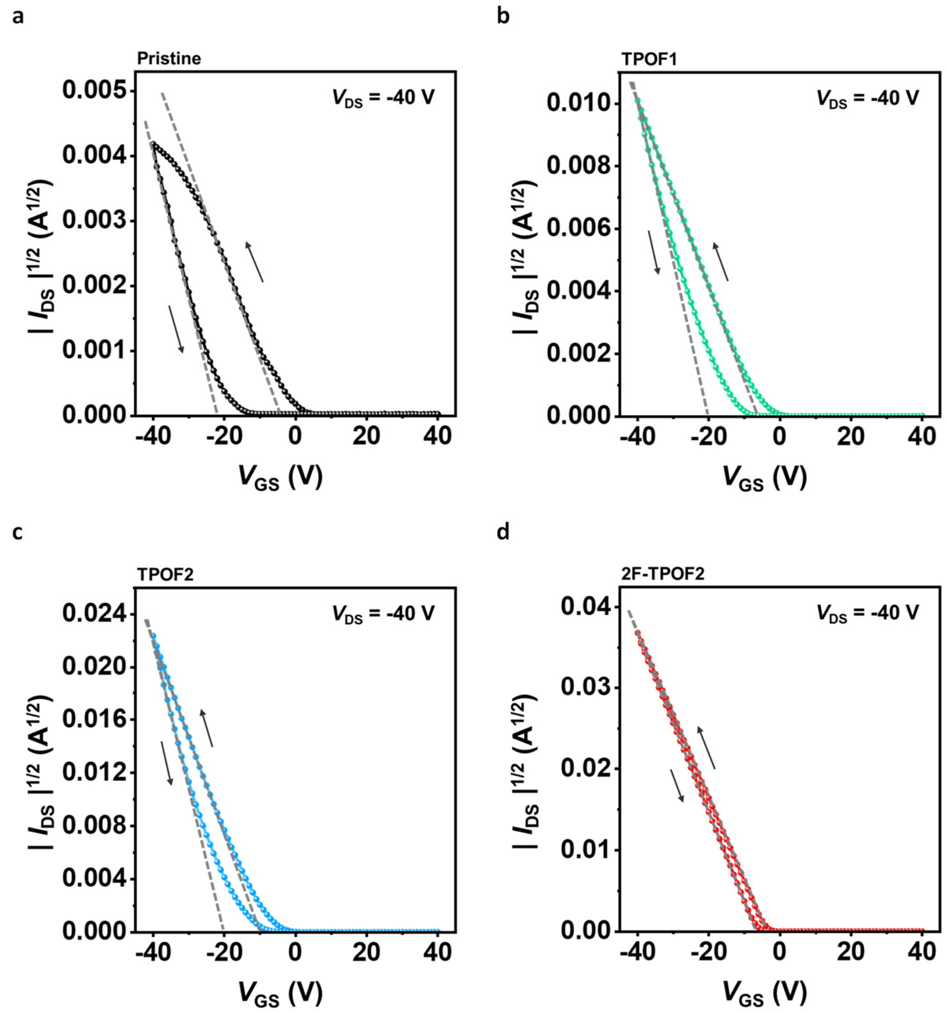

Figure S29. Transfer characteristics ($V_G - \sqrt{|I_{DS}|}$) of PEA_2SnI_4 FETs with and without molecular interlayers in saturation regime: **a** pristine PEA_2SnI_4, **b** PEA_2SnI_4 with TPOF1, **c** PEA_2SnI_4 with TPOF2, and **d** PEA_2SnI_4 with 2F-TPOF2. The grey dashed lines indicate the slope for the calculation of claimed mobility (μ_{claimed}).

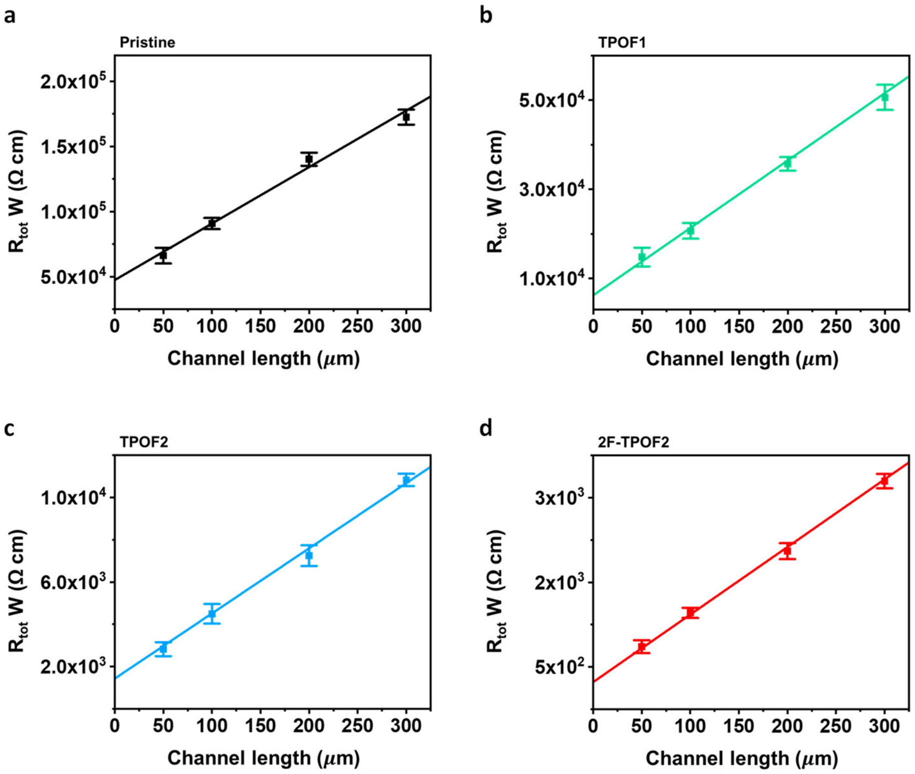

Figure S30. Transmission-line method for contact resistance calculation of PEA_2SnI_4 FETs with and without molecular interlayers. Contact resistance values are determined by the y-axis intercepts and the drain currents, collected from the linear regime (V_{GS} = -40 V and V_{DS} = -5 V): **a** pristine PEA_2SnI_4, **b** PEA_2SnI_4 with TPOF1, **c** PEA_2SnI_4 with TPOF2, and **d** PEA_2SnI_4 with 2F-TPOF2.

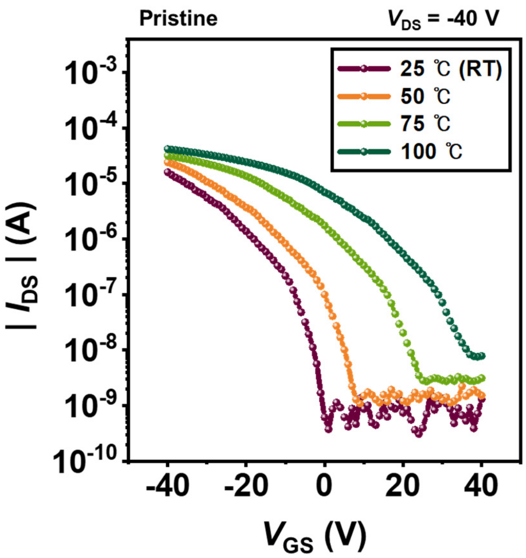

Figure S31. Temperature-dependent transfer curves measured on pristine PEA_2SnI_4 FET at various temperatures up to 100 °C.

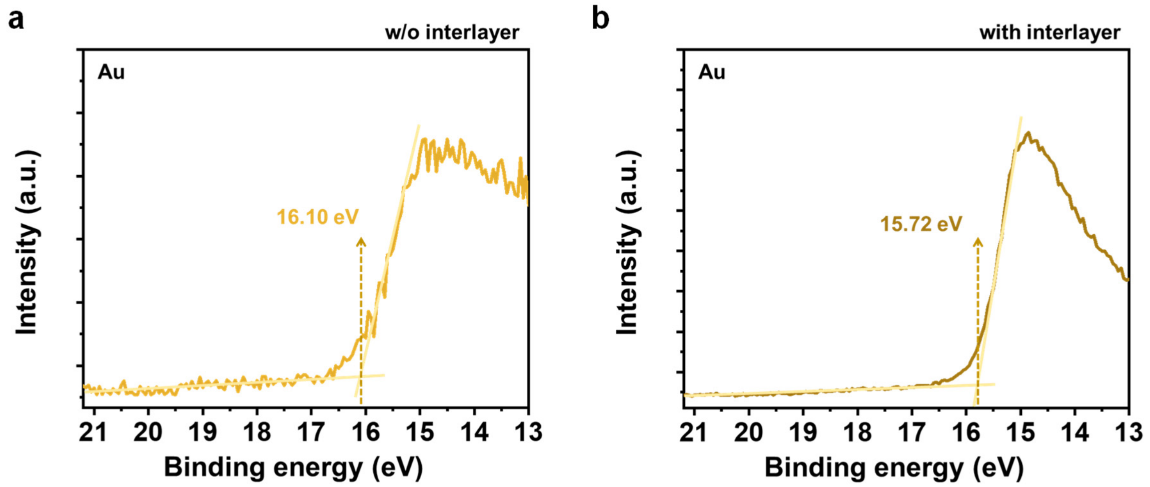

Figure S32. Ultraviolet photoelectron spectroscopy (UPS) binding energy profile representing the secondary electron cut-off energy associated with the work function (φ) of gold electrode **a** with and **b** without molecular interlayer (2F-TPOF2).

Table S1. Experimentally estimated energy levels of the designed molecules.

	λ_{edge} (nm)	E_{gap} (eV)^{opt}	E_{gap} (eV)^{cv}	E_{HOMO} (eV)^{cv}	E_{LUMO} (eV)^{cv}	E_{HOMO} (eV)$^{cv\&opt}$
**TPOF1**	441	2.81	2.37	-5.97	-3.60	-6.41
**TPOF2**	450	2.76	2.36	-5.98	-3.62	-6.38
**2F-TPOF2**	451	2.75	2.36	-5.99	-3.63	-6.38

λ_{edge}(nm) and E_{gap} (eV)^{opt} are estimated by UV-visible spectra.
E_{HOMO} (eV)^{cv}, E_{LUMO} (eV)^{cv}, and E_{gap} (eV)^{cv} are estimated by cyclic voltammetry data.
E_{HOMO} (eV)$^{cv\&opt}$ are estimated by E_{LUMO} (eV)^{cv} and E_{gap} (eV)^{opt}.

Table S2. Device performance of the $(PEA)_2SnI_4$ TFTs using various passivation layers from the forward transfer characteristics.

	Maximum $\mu_{claimed}$ [a)] [$cm^2\ V^{-1}\ s^{-1}$]	Average $\mu_{claimed}$ [a)] [$cm^2\ V^{-1}\ s^{-1}$]	V_{on} [V] [b)]	I_{on}/I_{off}	SS [V dec^{-1}] [c)]
**Pristine**	0.29	0.27 ± 0.04	8.7 ± 1.95	1.12×10^5	2.84 ± 0.02
**TPOF1**	2.26	2.23 ± 0.02	4.5 ± 0.81	1.37×10^6	1.93 ± 0.02
**TPOF2**	6.65	6.61 ± 0.03	2.6 ± 0.46	7.87×10^6	1.46 ± 0.01
**2F-TPOF2**	14.08	14.02 ± 0.03	1.1 ± 0.20	1.32×10^7	0.89 ± 0.01

a) $\mu_{claimed}$: claimed carrier mobility
b) V_{on} : turn-on voltage
c) SS: subthreshold swing

Table S3. Summary of the device parameters for previously reported 2D Sn metal halide perovskite FETs.

Semiconductor / Dielectric, thickness (nm)	Structure[1)]	Channel W / L (um)	Claimed field effect mobility ($cm^2\ V^{-1}\ s^{-1}$) / extraction method	$I_{on/off}$	*SS* ($V\ dec^{-1}$)	V_{th} (V)	Ref
**PEA_2SnI_4 / SiO_2, 300**	**BGTC**	**1500 / 100**	**14.08 / saturation regime**	**1.32×10^7**	**0.89**	**-3.4**	**This Work**
PEA_2SnI_4 / SiO_2, 300	BGBC	1000 / 160	0.68 / saturation regime	1.6×10^5	-	10	[9]
BA_2SnI_4 / PVA & CL-PVP, -	BGTC	1000 / 50	1.52 / saturation regime	1.5×10^2	22	24	[10]
PEA_2SnI_4 / PVA & CL-PVP, -	BGTC	1000 / 50	0.25 / -	10^4	5.6	22	[11]
PEA_2SnI_4 / PVA & CL-PVP, -	BGTC	1000 / 50	0.44 / -	10^4	6.1	13	[11]
$(TT)_2SnI_4$ / SiO_2, 300	BGTC	-	9.35 / saturation regime	8×10^5	5.4	-20	[12]
$BA_2SnI_{4-x}Cl_x$ / SiO_2, 300	BGBC	- / 20	0.1 / linear regime	-	-	-	[13]
PEA_2SnI_4 / SiO_2, 100	BGTC	1000 / 150	4.2 / saturation regime	6.7×10^5	1.9	-	[14]
PEA_2SnI_4 / SiO_2, 100	BGTC	1000 / 200	3.51 / saturation regime	3.4×10^6	0.8	-	[15]
PEA_2SnI_4 / CYTOP, 530	TCTG	2000 / 95	12.0 / saturation regime	2.6×10^6	0.8	-22	[16]
$BDASnI_4$ / PVA & CL-PVP, -	BGTC	1000 / 50	0.58 / saturation regime	5.0×10^4	6.0	6.99	[17]
PEA_2SnI_4 / SiO_2, 100	BGTC	1000 / 100	2.13 / -	-	0.77	-	[18]
PEA_2SnI_4 / SiO_2, 100	BGTC	1000 / 100	2.61 / saturation regime	9.0×10^6	0.5	7	[19]
PEA_2SnI_4 / PVA & CL-PVP, -	BGTC	1000 / 50	0.31 / saturation regime	5×10^3	9.4	18	[20]
PEA_2SnI_4 / PVA, 360 & CL-PVP, 30	BGTC	1000 / 50	0.33 / saturation regime	1×10^3	7.9	21	[21]
$(4TM)_2SnI_4$ / SiO_2, 300	BGTC	2880 / 40	2.32 / saturation regime	6×10^5	10	-20	[22]
PEA_2SnI_4 / SiO_2, 100	BGTC	1000 / 150	3.8 / saturation regime	7.4×10^5	2.1	-	[23]
PEA_2SnI_4 / SiO_2, 100	BGTC	1000 / 100	1.51 / saturation regime	3.1×10^5	2.1	25	[24]

PEA_2SnI_4 / SiO_2, 300 & polyimide, 80	BGBC	1000 / 105	2.6 / saturation regime 1.7 / linear regime	1×10^6	-	-	[25]
PEA_2SnI_4 / PVA, 100	BGBC	1000 / 28	0.62 /saturation regime	3×10^4	-	-30	[26]
PEA_2SnI_4 / SiO_2, 100	BGTC	1000 / 50	2.96 / saturation regime	2.69×10^4	-	-27.5	[27]
PEA_2SnI_4 / SiO_2, 270	BGTC	1000 / 150	0.17 / saturation regime	2×10^5	2.2	30.9	[28]
PEA_2SnI_4 / SiO_2, 200	BGTC	1000 / 50	1.45 / saturation regime	2.9×10^5	2.6	11.3	[29]
PEA_2SnI_4 / SiO_2, 300	BGTC	1000 / 80	0.7 / saturation regime	6.1×10^4	2.9	9	[30]
$BDASnI_4$ / SiO_2, 300	BGTC	2000 / 50	1.61 / saturation regime	4.7×10^6	-	-	[31]
$(HA)_2SnI_4$ / SiO_2, 300	BGTC	1000 / 80	0.009 / saturation regime	10^3	-	-	[32]
PEA_2SnI_4 / SiO_2, 300	BGTC	1000 / 100	4.25 / saturation regime	10^6	-	-	[33]

[1)]BGTC, TCTG and BGBC represent bottom-gate top-contact, top-contact top-gate and bottom-gate bottom-contact, respectively.

Table S4. Detailed parameters for the reliability factor (r_{sat}) and effective mobility ($\mu_{effective}$) of the $(PEA)_2SnI_4$ TFTs using various passivation layers under the forward scan in saturation regime.

	$\mu_{claimed}$ [a) [cm^2 V^{-1} s^{-1}]	r_{sat} [b)]	$\mu_{effective}$ [c)] [cm^2 V^{-1} s^{-1}]
**Pristine**	0.29	46.1 %	0.13
**TPOF1**	2.26	69.9 %	1.58
**TPOF2**	6.65	50.7 %	3.37
**2F-TPOF2**	14.08	80.4 %	11.32

[a)] $\mu_{claimed}$: claimed carrier mobility
[b)] r_{sat}: measurement reliability factor
[c)] $\mu_{effective}$: effective carrier mobility (= $r_{sat} \times \mu_{claimed}$)

References in Supplementary Information

1. C. M. Whitaker, K. L. Kott, R. J. McMahon, *J. Org. Chem.* **1995**, 60, 3499.
2. M. M. Alam, B. Biswas, A. K. Nedeltchev, H. Han, A. D. Ranasinghe, P. K. Bhowmik, K. Goswami, *Polymers* **2019**, 11, 1141.
3. K. Pilgram, R. D. Skiles, J. Heterocycl. Chem. **1974**, 11, 777.
4. H. Guo, H. Zhang, C. Shen, D. Zhang, S. Liu, Y. Wu, W. -H. Zhu, *Angew. Chem. Int. Ed.* **2021**, 60, 2674.
5. R. Lin, K. Xiao, Z. Qin, Q. Han, C. Zhang, M .Wei, M. I. Saidaminov, Y. Gao, J. Xu, M. Xiao, A. Li, J. Zhu, E. H. Sargent, H. Tan, *Nat. Energy* **2019**, 4, 864.
6. B. -B. Yu, Z. Chen, Y. Zhu, Y. Wang, B. Han, G. Chen, X. Zhang, Z. Du, Z. He, *Adv. Mater.* **2021**, 33, 2102055.
7. S. M. Sze, K. K. Ng, *Physics of Semiconductor Devices*, 3rd edn, John wiley & sons, 2007.
8. H. H. Choi, K. Cho, C. D. Frisbie, H. Sirringhaus, V. Podzorov, *Nat. Mater* **2018**, 17, 2.
9. Y. Liu, P. -A, Chen, X. Qiu, J. Guo, J. Xia, H. Wei, H. Xie, S. Hou, M. He, X. Wang, Z. Zeng, L. Jiang, L. Liao, Y. Hu, *iScience* **2022**, 25, 104109.
10. X. Liu, H. Ji, L. Li, F. Zhang, J. Guo, L. Qin, Z. Lou, D. Li, Y. Hu, Y. Hou, F. Teng, *ACS Appl. Mater. Interfaces* **2022**, 14, 50401.
11. F. Zhang, Q. Zhang, X. Liu, L. Qin, Y. Hu, Z. Lou, Y. Hou, F. Teng, *J. Mater. Chem. A* **2021**, 9, 22842.
12. A. Liang, Y. Gao, R. Asadpour, Z. Wei, B. P. Finkenauer, L. Jin, J. Yang, K. Wang, K. Chen, P. Liao, C. Zhu, L. Huang, B. W. Boudouris, M. A. Alam, L. Dou, *J. Am. Chem. Soc.* **2021**, 143, 15215.
13. J. Wang, H. Shen, W. Li, S. Wang, J. Li, D. Li, *Adv. Sci.* **2019**, 6, 1802019.
14. H. Zhu, A. Liu, T. Zou, H. Jung, S. Heo, Y. -Y. Noh, *Mater. Today Energy* **2021**, 21, 100722.
15. H. Zhu, A. Liu, K. I. Shim, J. Hong, J. W. Han, Y. -Y. Noh, *Adv. Mater.* **2020**, 32, 2002717.
16. T. Matsushima, S. Hwang, A. S. D. Sandanayaka, C. Qin, S. Terakawa, T. Fujihara, M. Yahiro, C. Adachi, *Adv. Mater.* **2016**, 24, 10275.
17. H. Ji, X. Liu, L. Li, F. Zhang, L. Qin, Z. Lou, D. Li, Y. Hu, Y. Hou, F. Teng, *J. Mater. Chem. A* **2023** 11, 7767.
18. J. -Y. Go, H. Zhu, Y. Reo, H. Kim, A. Liu, Y. -Y. Noh, *ACS Appl. Mater. Interfaces* **2022**, 14, 9363.
19. Y. Reo, H. Zhu, J. -Y. Go, K. I. Shim, A. Liu, T. Zou, H. Jung, H. Kim, J. Hong, J. W. Han, Y. -Y. Noh, *Chem. Mater.* **2021**, 33, 2498.
20. F. Zhang, Q. Zhang, X. Liu, Y. Hu, Z. Lou, Y. Hou, F. Teng, *ACS Appl. Mater. Interfaces* **2021**, 13, 24272.
21. F. Zhang, H. Zhang, L. Zhu, L. Qin, Y. Wang, Y. Hu, Z. Lou, Y. Hou, F. Teng, *J. Mater. Chem. C* **2019,** 7, 4004.
22. Y. Gao, Z. Wei, P. Yoo, E. Shi, M. Zeller, C. Zhu, P. Liao, L. Dou, *J. Am. Chem. Soc.* **2019**, 141, 15577.
23. H. Zhu, A. Liu, H. Kim, J. Hong, J. -Y. Go, Y. -Y. Noh, *Chem. Mater.* **2020**, 33, 1174.
24. H. Zhu, A. Liu, H. L. Luque, H. Sun, D. Ji, Y. -Y. Noh, *ACS Nano* **2019**, 13, 3971.
25. D. B. Mitzi, C. D. Dimitrakopoulos, J. Rosner, D. R. Medeiros, Z. Xu, C. Noyan, *Adv. Mater.* **2002**, 14, 1772.

26. C. R. Kagan, D. B. Mitzi, C. D. Dimitrakopoulos, *Science* **1999**, 286, 945.
27. I. -H. Chao, Y. -T. Yang, M. -H. Yu, C. -H. Chen, C. -H. Liao, B. -H. Lin, I. -C. Ni, W. -C. Chen, A. W. Y. Ho-Baillie, C. -C. Chueh, *Small* **2023**, 19, 2207034.
28. Y. Kim, J. Woo, Y. -K. Jung, H. Ahn, I. Kim, Y. Reo, H. Lim, C. Lee, J. Lee, Y. Kim, H. Choi, M. -H. Lee, J. Lee, S. D. Stranks, H. Sirringhaus, Y. -Y. Noh, K. Kang, T. Lee, *ACS Energy Lett.* **2024**, 9, 1725.
29. J. -H. Cho, J. -Y. Go, T. T. Bui, S. Mun, Y. Kim, K. Ahn, Y. -Y. Noh, M. -G. Kim, *Advanced Electronic Materials* **2023,** 9, 2201014.
30. S. Wang, K. Bidinakis, C. Haese, F. H. Hasenburg, O. Yildiz, Z. Ling, S. Frisch, M. Kivala, R. Graf, P. W. M. Blom, S. A. L. Weber, W. Pisula, T. Marszalek, *Small* **2023**, 19, 2207426.
31. X. Qui, J. Xia, Y. Liu, P. -A. Chen, L. Huang, H. Wei, J. Ding, Z. Gong, X. Zeng, C. Peng, C. Chen, X. Wang, L. Jiang, L. Liao, Y. Hu, *Adv. Mater.* **2023**, 35, 2305648.
32. S. Wang, S. Kalyanasundaram, L. Gao, Z. Ling, Z. Zhou, M. Bonn, P. W. M. Blom, H. I. Wang, W. Pisula, T. Marszalek, *Materials Horizons.* **2024**, 11, 1177-1187.
33. Y. Reo, T. Choi, J. -Y. Go, S. Jeon, B. Lim, H. Zhu, A. Liu, Y. -Y. Noh, *ACS Energy Lett.* **2023**, 8, 3088.
